# The Comparison of Expressed Candidate Secreted Proteins from Two Arbuscular Mycorrhizal Fungi Unravels Common and Specific Molecular Tools to Invade Different Host Plants

**DOI:** 10.3389/fpls.2017.00124

**Published:** 2017-02-07

**Authors:** Laurent Kamel, Nianwu Tang, Mathilde Malbreil, Hélène San Clemente, Morgane Le Marquer, Christophe Roux, Nicolas Frei dit Frey

**Affiliations:** ^1^Laboratoire de Recherche en Sciences Végétales, Université Paul Sabatier - Université de Toulouse, Centre National de la Recherche Scientifique Castanet-Tolosan, France; ^2^Agronutrition, Laboratoire de Biotechnologies Labege, France

**Keywords:** Glomeromycota, secretome, comparative transcriptomics, effectors, symbiosis

## Abstract

Arbuscular mycorrhizal fungi (AMF), belonging to the fungal phylum Glomeromycota, form mutualistic symbioses with roots of almost 80% of land plants. The release of genomic data from the ubiquitous AMF *Rhizophagus irregularis* revealed that this species possesses a large set of putative secreted proteins (RiSPs) that could be of major importance for establishing the symbiosis. In the present study, we aimed to identify SPs involved in the establishment of AM symbiosis based on comparative gene expression analyses. We first curated the secretome of the *R. irregularis* DAOM 197198 strain based on two available genomic assemblies. Then we analyzed the expression patterns of the putative RiSPs obtained from the fungus in symbiotic association with three phylogenetically distant host plants—a monocot, a dicot and a liverwort—in comparison with non-symbiotic stages. We found that 33 out of 84 RiSPs induced *in planta* were commonly up-regulated in these three hosts. Most of these common RiSPs are small proteins of unknown function that may represent putative host non-specific effector proteins. We further investigated the expressed secretome of *Gigaspora rosea*, an AM fungal species phylogenetically distant from *R. irregularis*. *G. rosea* also presents original symbiotic features, a narrower host spectrum and a restrictive geographic distribution compared to *R. irregularis*. Interestingly, when analyzing up-regulated *G. rosea* SPs (GrSPs) in different hosts, a higher ratio of host-specific GrSPs was found compared to RiSPs. Such difference of expression patterns may mirror the restrained host spectrum of *G. rosea* compared to *R. irregularis*. Finally, we identified a set of conserved SPs, commonly up-regulated by both fungi in all hosts tested, that could correspond to common keys of AMF to colonize host plants. Our data thus highlight the specificities of two distant AM fungi and help in understanding their conserved and specific strategies to invade different hosts.

## Introduction

Arbuscular Mycorrhizal fungi (AMF) are soil fungi belonging to the Glomeromycota, a basal phylogenetic lineage of fungi (Schüβler et al., 2001). All species of this clade are obligate mutualistic symbionts, mainly associated with plant roots (Smith and Read, 2010). AMF have aseptated hyphae and form spores accumulating hundreds to thousands of nuclei without any characterized mononuclear stage (Bécard and Pfeffer, 1993). As obligate biotrophs, AMF cannot be cultivated axenically and can only be propagated with plant roots in pot culture or *in vitro* on root organ culture (Bécard and Fortin, 1988). Due to these biological features and the absence of transformation protocol, their genetic structure is poorly documented. Transcriptomic and genomic approaches are useful tools to investigate their intimate biology (Tisserant et al., 2012, 2013; Lin et al., 2014). Interaction of AMF and host roots is triggered by signals that are exchanged prior to contact. AMF perceive plant exuded compounds, including the phytohormone strigolactones (Akiyama et al., 2005; Besserer et al., 2006), that modulate their metabolism and stimulate hyphal branching (Besserer et al., 2006, 2008), leading to higher probability of physical contact between the fungus and the plant root. After penetration, the fungus grows intra- and intercellularly and develops in host cells highly branched cell structures called arbuscules where nutrient exchanges occur between the two partners. Once the symbiosis is established, AMF produce a profuse mycelium outside of the root (ExtraRadical Mycelium or ERM). The ERM has a great implication in symbiotic physiology as it is involved in soil water and mineral foraging (Marschner and Dell, 1994; Wright et al., 1998). It is also crucial for fungal propagation through sporogenesis and colonization of new host roots.

Many questions remain unresolved about the molecular mechanisms governing AMF—plant interaction. A major issue concerns the broad host spectrum of these fungi: It was estimated that up to 80% of land plant species and 90% of Spermatophyta associate with AM fungi (Wang and Qiu, 2006; Smith and Read, 2010). As shown by inoculation assays in controlled condition or barcoding approaches in environmental samples (Öpik et al., 2010), each fungal species can be hosted by a large diversity of plant species. Usually, colonization of a host plant by pathogenic fungi encompasses intense cell signaling events that precede the activation of plant immunity. Surface or intracellular fungal molecules (Microbe Associated Molecular Patterns—MAMPs) or molecules released following host-degradation (Damaged Associated Molecular Patterns—DAMPs) are recognized by host cell receptors, thus triggering reactive oxygen species production, cell wall reinforcement, and secretion of antimicrobial compounds (Boller and Felix, 2009; Wu and Zhou, 2013). Hormone production (mainly salicylic acid, jasmonic acid, and ethylene) is also stimulated and promotes subsequent defense signaling, locally, or systemically (Bari and Jones, 2009). In order to overcome the plant defense, microbes limit their production of M(D)AMPs and secrete proteins, called effectors, that interfere with plant immunity. This strategy has been reported for many prokaryotic and eukaryotic microbial pathogens (Galán et al., 2014; Rovenich et al., 2014) and strong evidences support similar mechanisms in mutualistic interactions (Kloppholz et al., 2011; Plett et al., 2011; Okazaki et al., 2013; Yasuda et al., 2016). During AMF-plant interactions, non-self recognition should induce plant defenses thus limiting root colonization. Previous results argue that AMF have developed mechanisms to remain largely undetected by the plant defense, allowing their stealth growth in the roots. For example the low number of genes encoding Glycosyl hydrolases found in the genome of *Rhizophagus irregularis* could result in a low production of DAMPs (Tisserant et al., 2013; Lin et al., 2014).

The uniqueness of AMF to interact with a large range of host plants explains the interest on their effector catalog over the last few years. A first work provided evidence that *R. irregularis* secretes a small protein (SP7) that is translocated to the plant nucleus and facilitates the establishment of the interaction by repressing the activity of ERF19, a transcription factor mediating plant defenses (Kloppholz et al., 2011). More recently, transcriptomic approaches identified a second putative secreted protein, SIS1, whose expression is up-regulated during pre- and symbiotic stages. SIS1 was required for host colonization as well as arbuscular formation using silencing approaches (Tsuzuki et al., 2016). The release of genomic data from *R. irregularis* revealed the presence of hundreds of putative secreted proteins (SPs—Tisserant et al., 2013; Lin et al., 2014). In a kingdom-wide comparison of fungal secreted proteins, it was shown that *R. irregularis*, as other fungal mutualists, encodes more small secreted proteins than saprotrophs and necrotrophs (Kim et al., 2016) and a very low number of CAZymes (Tisserant et al., 2012, 2013; Kim et al., 2016). Recently, additional gene repertoires have been published, broadening the field of investigation: From *R. clarus* (Sędzielewska Toro and Brachmann, 2016), a species closely related to *R. irregularis*, and from *Gigaspora margarita* (Salvioli et al., 2015) and *G. rosea* (Tang et al., 2016). *Gigaspora* spp. belong to the order Diversisporales, phylogenetically distant from Glomerales that includes *R. irregularis* (Schüβler et al., 2001). Diversisporales and Glomerales present distinct morphological, ecological and biological features. *G. rosea* forms larger hyphae than those of *R. irregularis* and develops arbuscules that have a different morphology (Parniske, 2008). *R. irregularis* is an ubiquitous AMF observed in a wide range of hosts (Öpik et al., 2006; Börstler et al., 2008), while *G. rosea* was not reported so far in all continents (Jansa et al., 2002; Öpik et al., 2010) and several reports suggest that its host spectrum is narrower than *R. irregularis* (Russell and Bulman, 2005; Sýkorová et al., 2007). The non-homogeneous mycorrhizal responses induced on diverse plants by Glomerales and Diversisporales suggest host selectivities by these fungi (Hong et al., 2012; de Novais et al., 2014; Mensah et al., 2015). As an illustration, it was observed significant differences regarding host phosphate uptake, growth, and/or reproduction of flax, tomato, and barrel medic when associated with *G. rosea* compared to *R. irregularis* (Smith et al., 2003). Comparison of expressed secretomes from these two AMF is hence a way to define the convergent/divergent strategies for the establishment of the symbiotic interface. SPs can indeed be involved in mechanisms as various as fungal cell wall remodeling (Ene et al., 2015), substrate degradation (Bouws et al., 2008), nutrient recruitment from the host interfaces (Fernandez et al., 2014), and repression of host immunity (Krijger et al., 2014).

Our project aimed first at investigating whether the same set of SPs is consistently expressed by an AMF when colonizing different hosts. It also aimed at evaluating to which extent SPs, either involved in fungal cell processes (nutrition, cell wall formation, and modification) or in modulating plant immune responses (effectors) are conserved among AMF. We first generated a comprehensive list of putative SPs by applying a bioinformatic pipeline on the two independently generated genome assemblies of the same strain (Tisserant et al., 2013; Lin et al., 2014). Then, we investigated the expression profiles of *R. irregularis* SPs (RiSPs) obtained in association with three distant plant hosts. Finally we compared RiSP sequences and expression patterns to those of SPs from *G. Rosea* (GrSPs). Our findings showed that specific classes of SPs were expressed at different fungal developemental stages explored in this study. Comparative SP expression patterns in different hosts highlighted that *R. irregularis* displayed a lesser ratio of host-specific secreted proteins compared to *G. rosea*. Finally, *R. irregularis* and *G. rosea* were found to share a small but nevertheless interesting set of SPs that are good candidate effectors targeting host conserved mechanisms.

## Materials and methods

### Production of fungal materials

*R. irregularis* DAOM 197198 and *Gigaspora rosea* DAOM 194757 spores and extraradical mycelium (ERM) produced on carrot root organ cultures (St-Arnaud et al., 1996) were purchased from Agronutrition (Labège, France). Spores were germinated and grown in liquid M medium (Bécard and Fortin, 1988), in the dark at 30°C with 2% CO_2_. All biological samples were produced in triplicates for sequencing. *R. irregularis* and *G. rosea* were treated during 48 h with control solution or GR24 (10^−6^ M final) respectively 2 and 5 days post germination. For the production of mycelium exposed to root exudates, spores were deposited on a cellophane membrane placed on *in vitro* carrot root organ culture, or on a cellophane membrane placed on solid M medium for Mock condition, for 14 days (*R. irregularis*) or 10 days (*G. rosea*) at 30°C and 2% CO_2_. Concerning the production of Intra Radical Mycelium (IRM), *Medicago truncatula* Gaertn “Jemalong” genotype A17 and *Brachypodium distachyon* genotype Bd21 were cultivated in association with *R. irregularis* DAOM 197198 during 5 weeks. For *Gigaspora rosea* DAOM 194757, a nursery system was used to increase mycorrhizal rates in the same time frame of assay. *M. truncatula* and *B. distachyon* plantlets were co-cultivated on Oil-Dri US special substrate (Damolin) in pot containing a leek plant cultivated since 3 months with *G. rosea* (growing conditions: 25°C, 16 h of day and 22°C, 8 h of night). After 3 weeks, plants were removed from the nursery system and individualized in single pots for 2 additional weeks of culture. Mycorrhizal rates were assessed using the grid intersection line system (Giovannetti and Mosse, 1980) and roots were sampled when mycorrhization reached a sufficient colonization rate. The plants produced as triplicate for experiments were mycorrhized at 59, 61, and 58% for *M. truncatula* and 39, 48, and 39% for *B. distachyon* by *R. irregularis*, respectively. The colonization rate of all triplicate plants mycorrhized by *G. rosea* reached over 80% (detailed informations at NCBI GEO portal GSE67911). Plants were grown with 16 h light (25°C)/8 h (22°C) cycles and fertilized twice a week with 0.5x Long Ashton solution.

*Lunularia cruciata* were collected in the Pyrenees Mountains (France). Gemmae were sterilized (Fonseca et al., 2006) and grown on KNOP medium (Reski and Abel, 1985) at 22°C with a 16 h photoperiod. *L. cruciata* was mycorrhized according to Fonseca et al. (2006). As the intergrid method used to assess mycorrhizal rate in roots was not appropriate, we checked by staining that the fungus highly colonized the central part of the thalli.

### RNA production and sequencing

Total RNA extraction and sequencing were performed according to Tisserant et al. (2013) for *R. irregularis* and Tang et al. (2016) for *G. rosea*. Apart from ERM of *G. rosea* where short paired- end sequencing reads were obtained from Illumina Miseq1000 protocols (2 × 151 bp), all libraries were obtained fromIllumina Hiseq2000 protocols (2 × 101 bp). Library constructions and sequencing were performed on the GeT-PlaGe facility (Toulouse, France), according to standard Illumina protocols. Data are available at NCBI GEO portal (GSE67906 and GSE67911) for *G. rosea*, and at NCBI Sequence Read Archive (SRR1027885) and at NCBI GEO portal (GSE67926) (—see details on libraries in Table S1A) for *R. irregularis*. Number of reads per libraries, representativeness of fungal reads in symbiotic tissues and variability of data are presented in Tables S1A, S5A for *R. irregularis* and *G. rosea* respectively.

### Bioinformatic analysis

GMAP analysis (Wu and Watanabe, 2005) was performed with the standard parameters between the two genomic assemblies of *R. irregularis* (Tisserant et al., 2013; Lin et al., 2014). When different gene definitions were present for the same locus, only ORFs supported by RNAseq data were selected and protein lacking a start or a stop codon were discarded. Because of a lack of complete coverage by RNAseq reads, SP7 (Kloppholz et al., 2011) was at first absent from the analysis. We therefore used the second assembly (Lin et al., 2014) that contains a SP7 gene definition (Rir018650) to recover this protein in the RiSPs set. In order to be easily identified in the study, this gene is not designated by a RiSP number but keeps its original name. SIS1, a previously characterized putative secreted protein that was described to be essential for mycorhizae establishment (Tsuzuki et al., 2016) is absent from our set of 872 RiSPs. Indeed, in our pipeline, a transmembrane domain was identified in the protein sequence. RiSPs and GrSPs were identified using the following pipeline with standard software parameters. SignalP3.0 (Bendtsen Dyrløv et al., 2004) allowed the identification of a signal peptide, TMHMM (Krogh et al., 2001), and Phobius (Käll et al., 2004) excluded proteins presenting a transmembrane domain. TargetP (Emanuelsson et al., 2007) was used to remove proteins with a mitochondrion targeting signal. We selected mature proteins as small as 15 amino acids after the cleavage of the peptide signal and discarded protein sequences with duplicates. No upper size limit was applied. This screen resulted in a list of 872 proteins for *R. irregularis* and 2633 for *G. rosea* (Tables S1C, S5C, respectively). In these repertoires, we searched for conserved domains by PFAM analysis (Finn et al., 2014), repeated motifs by T-reks analysis (Jorda and Kajava, 2009), Nuclear Localization by NLStradamus analysis (Nguyen Ba et al., 2009) and we scored disulfide-bond forming proteins among small cysteine-rich proteins (SCR) with Disulfind (Ceroni et al., 2006). Finally, Blastclust (Alva et al., 2016) was used to identify paralog groups of proteins. The MEME discovery tool (Bailey et al., 2009) was used to screen GrSPs and RiSPs displaying no PFAM domain in order to identify previously unknown motifs, present as internal repeats or displayed by several proteins. A first screen searching for short motifs (3 to 6 amino acids) was performed and identified a subgroup of SPs containing short internal repeats. These proteins were then excluded from the second screen focusing on SPs diplaying longer motifs (7 to 100 amino acids). Only motifs present at least three times and with an e-value < 10^−3^ were conserved. Motifs spanning almost entirely the sequence of tribe members were not conserved, since sequence similarity introduces a strong bias in motif identification. GPI anchor domains containing proteins are not considered as true secreted proteins and are thus not discussed in the present study. SCR proteins were described as proteins with less than 200 amino acids, containing at least two cysteines and with a 3% minimum Cysteine content. MAFFT (Katoh et al., 2002) was used to align SP7 with its relative SPs. For comparison, the 726 RiSPs predicted from Gloin1 gene models were compared to the previously identified *R. irregularis* SPs of the fungal secretome database (classes: SP, SP^3^, and SL—Choi et al., 2010). To identify putative RiSPs and GrSPs orthologs in other fungi, BLAST-P analyses were performed against the RefSeq with a e-value < 10^−5^ with exclusion of glomeromycota proteins (Altschul et al., 1990). The 220 *R. irregularis* and 64 *R. clarus* candidate effectors described in Sędzielewska Toro and Brachmann (2016) were compared to RiSPs through BLAST-P analysis; a e-value < 10^−5^ was selected to define putative orthologous proteins. *G. margarita* genes were analyzed through our pipeline to identify SPs with a similar approach and compared through BLAST-P analysis to GrSPs; a e-value < 10^−5^ was selected to define putative orthologous proteins. The same analysis was performed with *P. indica* (Zuccaro et al., 2011), *T. melanosporum* (Martin et al., 2010), and *L. bicolor* genes (Martin et al., 2008) for comparison with RiSPs. Search for isoforms in GrSPs was performed by reciprocal BLASTN (GrSPs against themselves with the following criteria: Identity>97%, query coverage>50%, query hit>10%) followed by sequence alignments using SeaView version 4 program for validation (Gouy et al., 2010).

### Gene expression and differential expression analysis

Raw sequence paired reads were trimmed using CLC Genomics workbench 8.0 suite (CLC Bio workbench, Qiagen, Aarhus, Denmark) based on Phred quality scores > 20 by removal of adapter Illumina primer, trimming end sequences of reads to limit the number of ambiguous nucleotides at 2, and discarding of reads shorter than 50 bp. Homogeneity of triplicates was defined by carrying out principal component analyses of samples according to CLC Genomic Workbench procedure and confirms the grouping of samples (Tables S1A, S5A for *R. irregularis* and *G. rosea* respectively). For each replicates, the correlation matrix (“normalized” version of the covariance matrix) was calculated to define the orthogonal eigenvectors of the first and second major principal components, showing a simplified version of the variability of data. For expression analyses, trimmed pair-ended reads were mapped onto the transcripts of 872 and 2633 SP genes of *R. irregularis* and *G. rosea*, respectively, using CLC Genomics workbench with stringent settings (similarity and length read mapping criteria at 98 and 95% respectively, maximum number of hits for a read on different genes limited to 10). We used the settings “one reference sequence per transcript” and “Maximum number of hits for a read = 10” to define unique and total reads mapping on each transcript, allowing the differenciation of expression patterns of close sequences. The mapped reads for each transcript were calculated and normalized as RPKM for calculating gene expression (reads per kilobase of transcripts per million reads mapped—Mortazavi et al., 2008). Intact and broken pairs were both counted as one. The RPKMs of each transcript in different conditions were compared using proportion-based test statistics (Baggerly et al., 2003) implemented in CLC genomic Workbench suite. This beta-binomial test compares the proportions of counts in a group of samples against those of another group of samples. Different weights are given to the samples, depending on their sizes (total counts). The weights are obtained by assuming a Beta distribution on the proportions in a group, and estimating these, along with the proportion of a binomial distribution, by the method of moments. The result is a weighted *t*-type test statistic. We then calculated False Discovery Rate (FDR) correction for multiple-hypothesis test (Benjamini and Hochberg, 1995). Only genes showing a difference of 10 reads between compared conditions were considered as significantly expressed. Genes were considered as differentially expressed when meeting the requirements of fold change ≥|2| and FDR ≤ 0.05. Extreme fold-change values (+/– 1,79769313486232E+308) are depicted as “+/–999999” in Tables S1, S5. RNAseq raw expressed data and calculated fold changes are depicted in Tables S1B, S5B for *R. irregularis* and *G. rosea* respectively.

## Results

### Definition of a consensus set of SPs of *Rhizophagus irregularis* DAOM 197198

**Figure 1:**
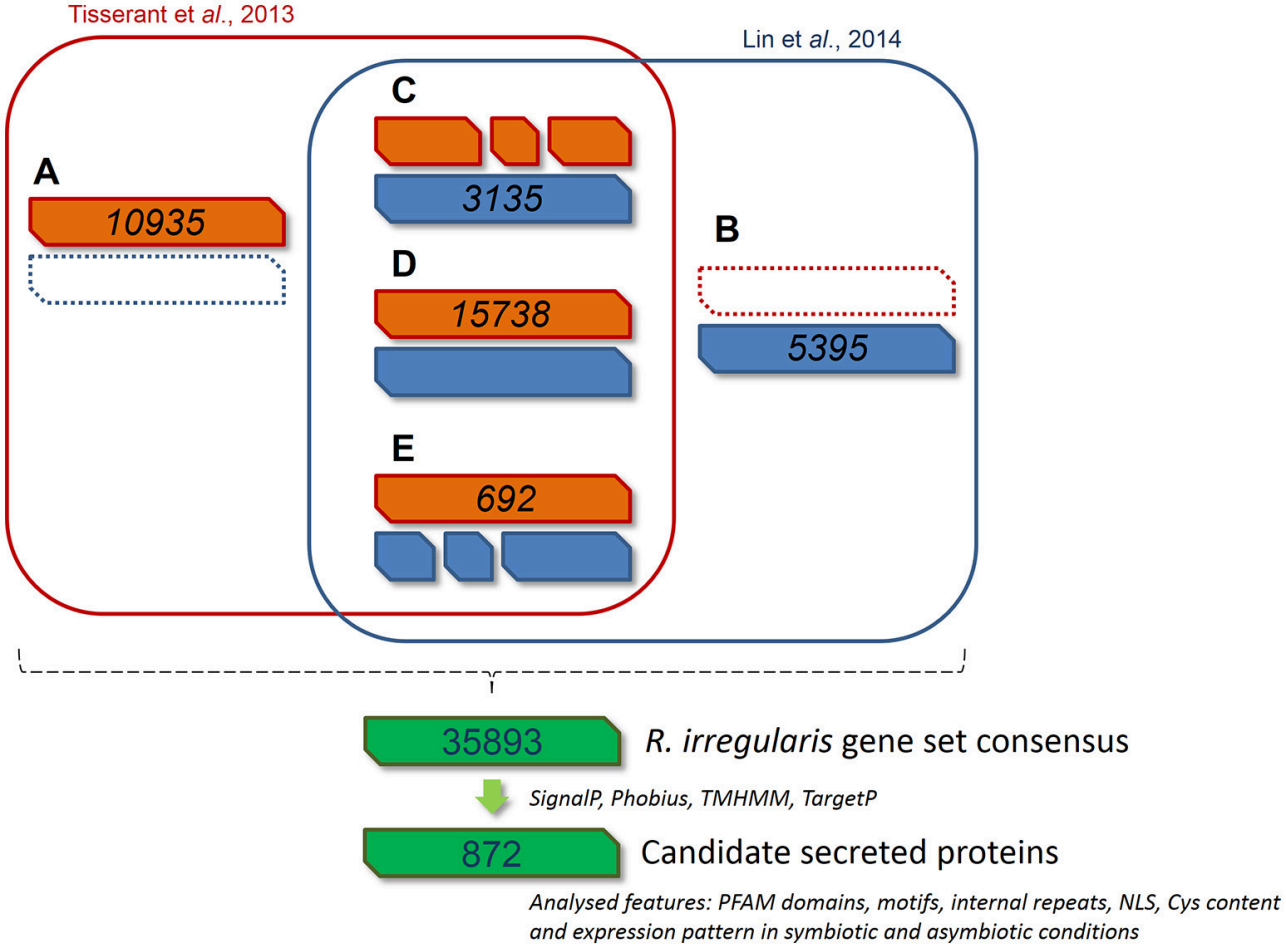
**Bioinformatic pipeline describing the process and the gene model screening for *R. irregularis* secretome prediction**. 30282 and 27300 gene models published by Tisserant et al. (2013) and Lin et al. (2014) respectively were compared through a GMAP analysis to generate a consensus gene set. Genes from classes **(A—E)** were pooled to constitute a new set of 35893 genes. **(A)** 10935 genes specific to Tisserant assembly. **(B)** 5395 genes specific to Lin assembly. **(C)** 3135 genes in Lin assembly that correspond to fragmented Tisserant ORFs. **(D)** 15738 genes in Tisserant assembly with a single map in Lin assembly. **(E)** 692 genes in Tisserant assembly that correspond to fragmented Lin ORFs. Secreted proteins were then identified as proteins with a signal peptide (SignalP 3.0), an absence of transmembrane domain (TMHMM, Phobius), and mitochondrion targeting (TargetP). Presence of PFAM domain, NLS, internal repeats, Cys content where then scored and expression pattern was analyzed in symbiotic and asymbiotic conditions. See methods for details.

Two independent genomic assemblies of the same strain of *R. irregularis* (DAOM 197198) were previously published (Tisserant et al., 2013; Lin et al., 2014). Due to incomplete assembly, the deduced catalogs of putative SPs from these two sets of data were partial and very few SPs were found totally identical by direct blast (Table S1C, see column B). In order to get a more robust global dataset, we performed a bidirectional Genomic mapping and alignment program (GMAP) analysis (Wu and Watanabe, 2005) of the two gene repertoires. GMAP allowed the identification of common genes (perfect match), genes unique to each dataset, and genes partly similar, i.e., truncated or differently defined in each gene repertoire. For the last two situations, ORFs covered by reads from RNAseq data were given preference (http://genome.jgi.doe.gov/Gloin1/Gloin1.home.html). Based on this approach, we obtained 35893 predicted genes. Using a specific pipeline to identify putative secreted proteins (Figure 1), we then defined a list of 872 secreted protein encoding genes (RiSPs) (Table S1C). This repertoire is larger than previously published ones from different assemblies and pipelines (376 SPs in Tisserant et al., 2013; 566 SPs in Lin et al., 2014; 475 SPs in Kim et al., 2016). This was expected because our input uses two different assemblies while only one was processed in the previous works. In the case of Kim et al. (2016), they also removed proteins containing nuclear localization signals. As noticed by Sędzielewska Toro and Brachmann (2016), there is a very high similarity with *R. clarus* available secreted proteins (Table S1C). We compared the SPs that we identified from the Gloin1 gene models (726 Gloin1-originating RiSPs) to already characterized *R. irregularis* Gloin1-SP identified in the Fungal Secretome Database (FSD; Choi et al., 2010; Kim et al., 2016). These proteins were classified in various categories according to their signal peptide predictions (SP, SP^3^, SL, Table S1C). Due to differences in pipeline definition (e.g.,: SPs containing a NLS are discarded in the FSD), 216 out of 726 of our Gloin1-originating RiSPs are absent in the FSD.

Within the 872 RiSPs, different sequence features were identified: Presence of PFAM domains, nuclear localization sequences (NLS), cysteine rich regions (SCRs), organization as repeat contain proteins (RCPs), and distribution in protein families (tribes). As mentionned in Table S1C (“Tribe” column), 772 RiSPs were unique proteins, 54 were present in tribes of two members (D1–27) and 46 are grouped in 13 tribes of at least three members. Among the 872 RiSPs, 184 proteins displayed a PFAM domain. As observed in previous studies (Tisserant et al., 2012, 2013; Kim et al., 2016), *R. irregularis* displays a very limited number of CAZymes (25 RiSPs). Several PFAM domains were found enriched when compared to the whole gene repertoire (Table S2), including several proteases such as subtilases, aspartic proteases, M28 peptidases. Proteins involved in cell wall modification (polysaccharide deacetylase) or interaction with lipids (ML domain) were also found enriched. A survey of RiSPs present in other beneficial fungi revealed a low number of conserved SPs (Table S1). 92 RiSPs were conserved in *P. indica, T. melanosporum* or *L. bicolor*. Fourty-two were present in all three fungi within which 40 display a PFAM domain (see below).

### Expression patterns of RiSPs

**Figure 2:**
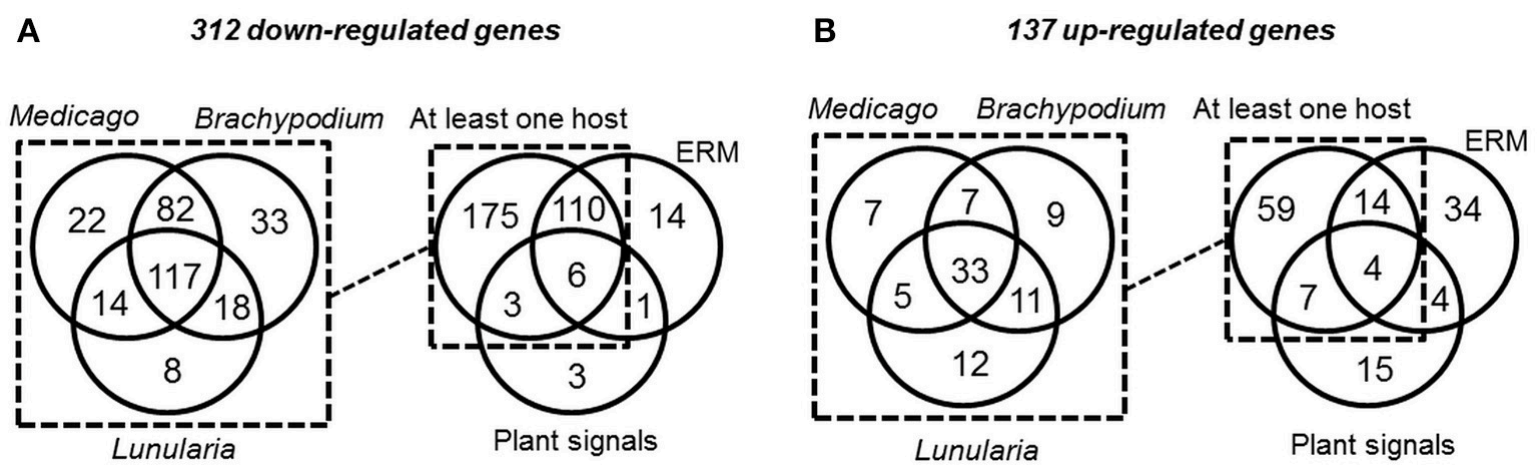
**Venn diagrams of *R. irregularis* SP (RiSPs) genes down- (A)** and up-regulated (B) in the different comparisons (see Table S1C for details). **(A**: Left panel**)** number of RiSPs down-regulated in the differents hosts. Right panel: RiSPs down-regulated in at least one host were pooled and compared to genes down-regulated in ERM or in germinating spores treated with plant signals (GR24 or plant exudates). **(B**: Left panel**)** number of RiSPs up-regulated in the differents hosts. Right panel: Genes up-regulated in at least one host were pooled and compared to genes down-regulated in ERM or in germinating spores treated with plant signals (GR24 or plant exudates).

As potentially important players in the interaction of AM fungi with their hosts, RiSP-coding genes are expected to be up-regulated in symbiotic conditions (Kloppholz et al., 2011). To analyse gene expression, RNAseq data were generated from various pre-symbiotic and symbiotic stages of fungal development (summarized in Figure S1; see methods). Briefly, spores of *R. irregularis* were germinated in liquid medium supplemented or not with root exudates or with GR24, a strigolactone synthetic analog. For symbiotic conditions, the fungus was cultivated in association with *L. cruciata, B. distachyon*, and *M. truncatula*. These three hosts—a liverwort, a monocot, and a dicot—belong to distant clades of the embryophytes, allowing to highlight host selectivity of SP-encoding genes. In addition to these three conditions of intraradical mycelium (IRM), extraradical mycelium (ERM) was also sampled from carrot root organ culture (Figure S1). Regarding global expression, 590 among the 872 RiSPs showed a transcriptional activity in at least one of the tested conditions (Tables S1B,C). At this stage, it is impossible to conclude whether the 282 SPs with no transcriptional activity are pseudogenes or just not detected/not expressed in these biological conditions. Around 70% of the expressed RiSPs presented a statistically robust (FDR < 0.05) up- or down-regulation pattern (137 and 312 respectively over a 2 fold change) in at least one experimental comparison (Figure 2). An important overlap of down regulated genes (116) was observed in IRM and ERM compared to the reference (germinating spores), representing 88% of the genes downregulated in the ERM (Figure 2A right panel). Independent biological samples were produced to perform RT-qPCR on ten IRM up-regulated genes in medicago. All of them validated the RNAseq data (Table S1F).

Considering the 137 up-regulated RiSPs, 84 are up-regulated in the IRM (Figure 2B right panel), among them 33 are shared between the three hosts (Figure 2B left panel, Table S1D), suggesting that *R. irregularis* produces a common set of RiSPs irrespectively of the colonized host. Among these 33 RiSPs, a majority (19) are short proteins (< 200 aa) and include 12 SCRs (Table S1D), two (RiSP646 and RiSP734) are predicted to possess a NLS and display repeated motifs and six present a PFAM domain. Conversely, one third of RiSPs (28 among 84) are specifically up-regulated in a single host: 7, 9, and 12 have a specific up-regulation in *M. truncatula, B. distachyon*, and *L. cruciata* respectively (Figure 2B left panel, Table S1E). Among these 28 RiSPs, 25 code for proteins without any predicted function and may represent candidate host specific effectors.

**Figure 3:**
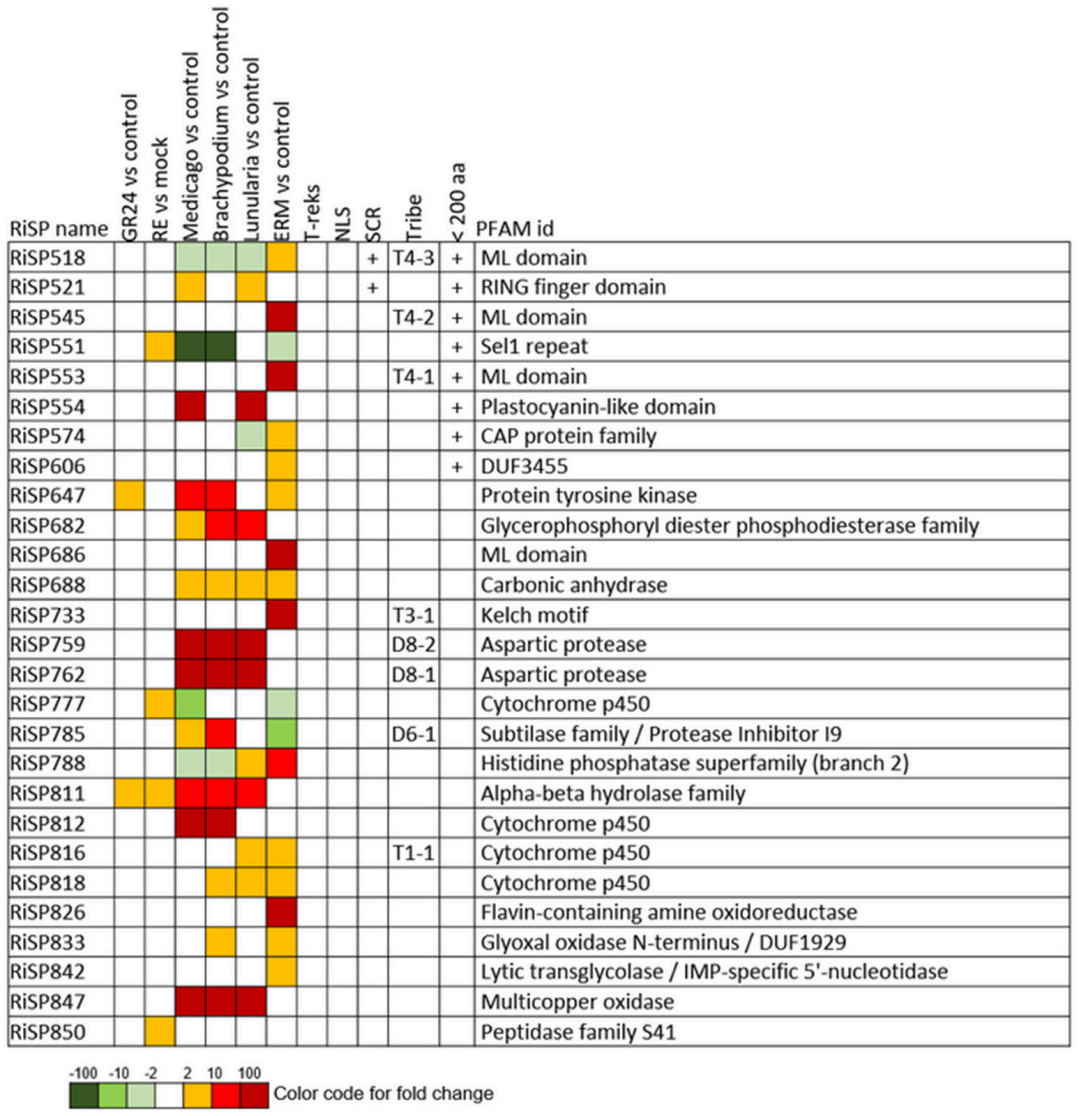
**RiSPs with a PFAM domain that are up-regulated in at least one comparison**. RE, root exudates; ERM, Extra-Radical Mycelium; NLS, Nuclear Localization Signal; SCR, Small Cysteine Rich.

When considering the predicted features and functions of the 137 *in planta* up-regulated RiSPs, 27 present a PFAM domain (Figure 3). These SPs display an enrichment for Cytochrome P450s and the lipid binding ML domain (Table S3). Three RiSPs are exclusively expressed *in planta*: The two aspartic proteases RiSP759 and RiSP762 and a protein with Cu-oxidase domains, RiSP847. This three RiSPs are conserved in *P. indica, T. melanosporum*, and *L. bicolor*, suggesting a shared role between beneficial fungi. RiSP847 is likely a laccase-like multicopper oxidase that may act as a lignin modifier, although secondary cell wall are weakly developped in epidermal and cortical root cell layers (Baldrian, 2006). RiSP833, only found up-regulated in *B. distachyon*, could be involved also in cell wall modification since glyoxal oxidase domain-containing proteins have been shown to degrade lignin (Whittaker et al., 1999). Additionally, RiSP574 is a CAP protein that has been described to fulfill different roles including cellular matrix remodeling, cell-cell adhesion, and also fungal virulence (Barrero et al., 2002; Schneiter and Di Pietro, 2013), that fits with its specific up-regulation in ERM. Similarly, RiSPs with lipid binding activities (ML domain) are also uniquely up-regulated in ERM. Four Cytochrome P450 with contrasted expression patterns may be involved in the production of fungal molecules or in cell detoxification required at different stage of development. Another protein of interest, induced by plant signals and also up-regulated in host roots encodes an alpha-beta hydrolase (RiSP811). Such proteins present a hydrophobic pocket and are known to play multiple roles in perception and cleavage of endogenous and xenobiotic compound (Carr and Ollis, 2009). A putatively secreted carbonic anhydrase (RiSP688) is up-regulated *in planta* but also in ERM. The role of secreted fungal carbonic anhydrases is yet unclear. However, their function seems shared between mutualistic fungi since this protein is also present in *P. indica, T. melanosporum* and *L. bicolor* (Table S1C). Intracellular isoforms were described to participate in the regulation of intercellular pH. For instance, Nce103, a *Saccharomyces cerevisiae* β-class of carbonic anhydrase, was shown to be important for the fungal growth under ambient air condition and play a role in ${\text{CO}}_{2}/{\text{HCO}}_{3}^{-}$ homeostasis (Götz et al., 1999; Amoroso et al., 2005; Elleuche and Poggeler, 2010). Secreted fungal carbonic anhydrases are supposed to have a role also in soil acidification (Thorley et al., 2015), thus allowing a better mineral acquisition in ERM.

**Figure 4:**
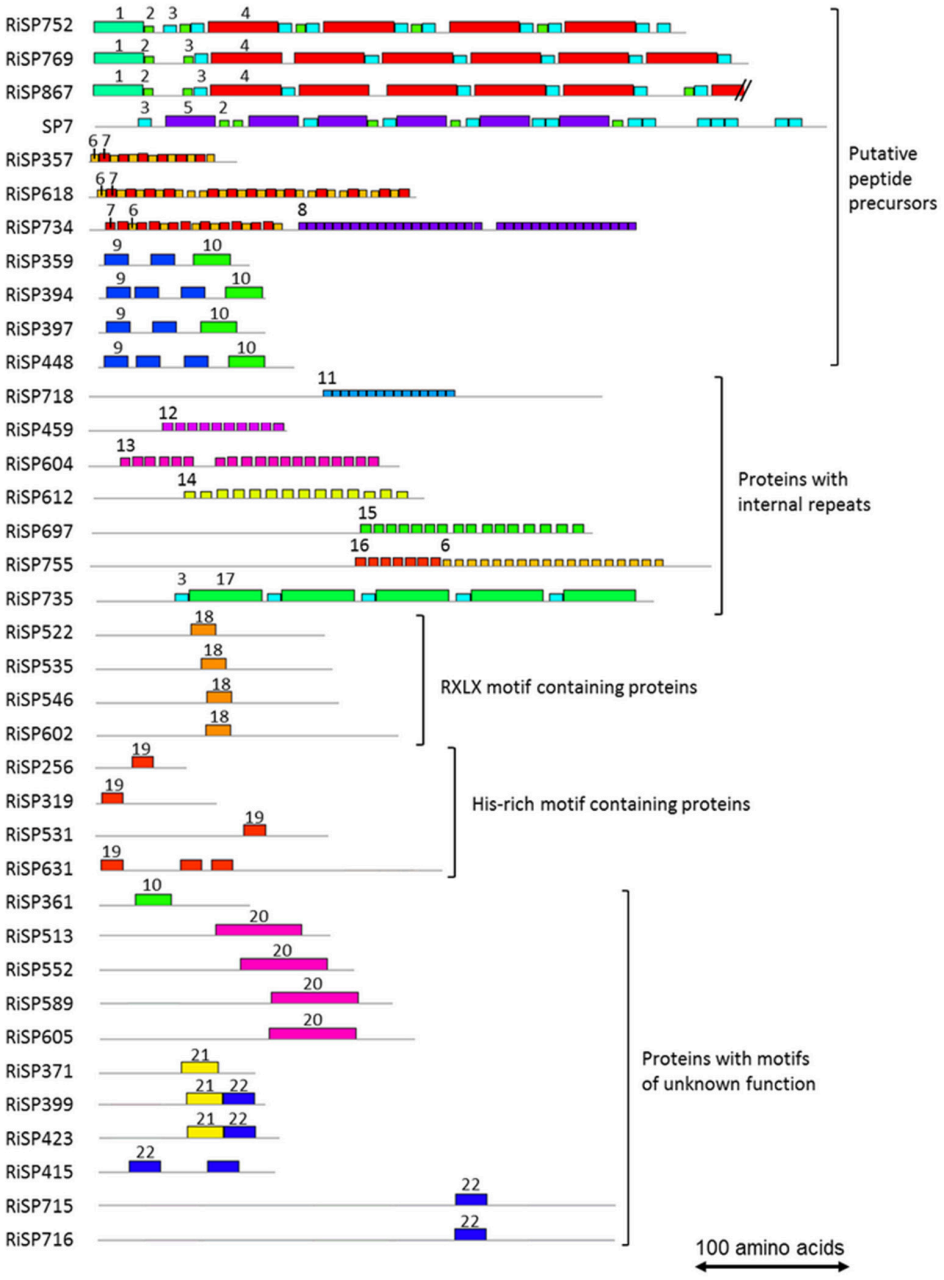
**Motifs identified in RiSPs through a MEME search**. Proteins are grouped according to their motif content, predicted maturation, or putative function. Motif sequences are listed in Figure S2. Each motif is illustrated by an unique color box and a number. To facilitate readability, numbers are mentioned only once for each proteins.

**Figure 5:**
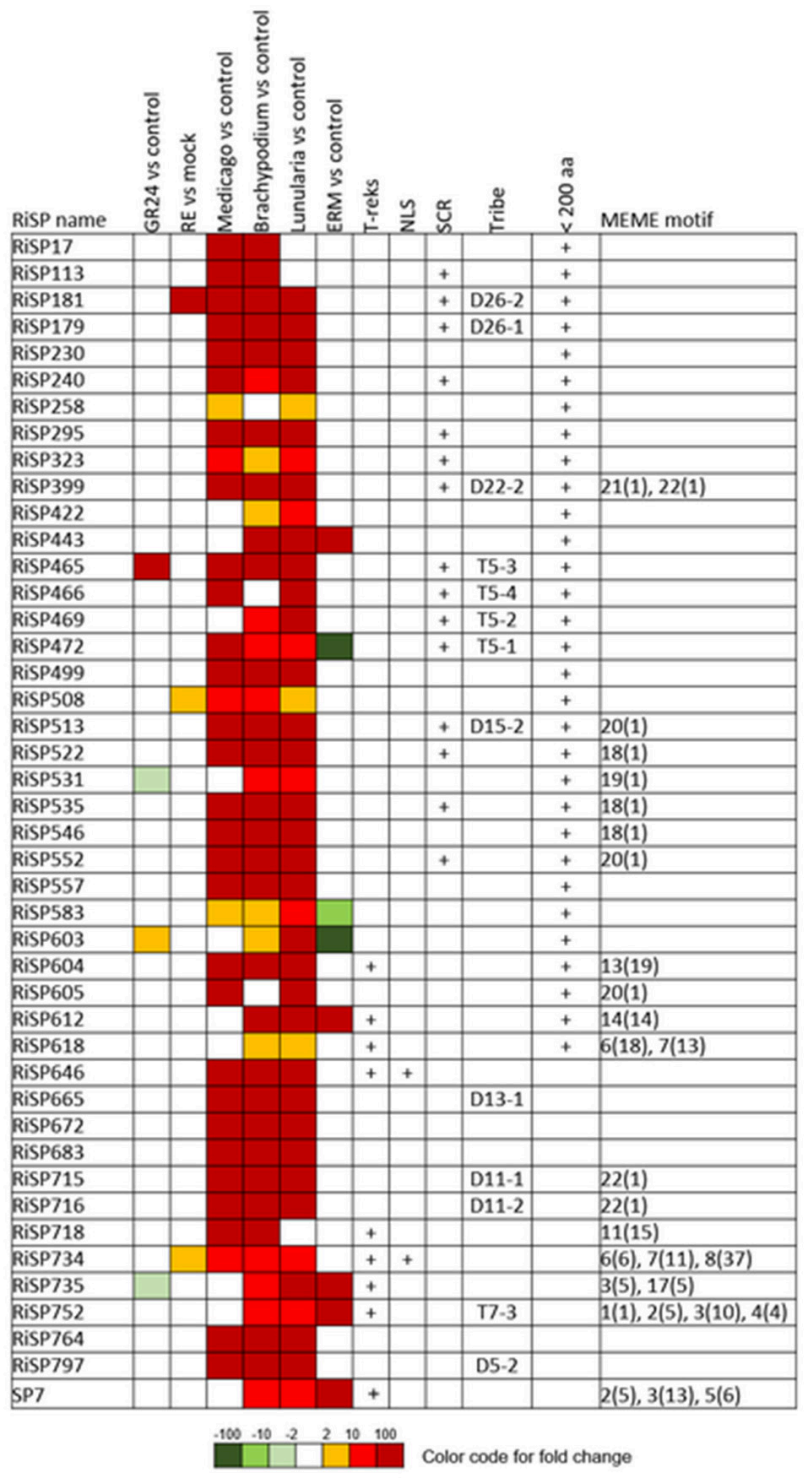
**RiSPs of unknown function preferentially up-regulated in symbiotic tissues**. MEME motifs are described by their number (Figure 4 and Figure S2) and their occurrence (value within brackets).

**Figure 6:**
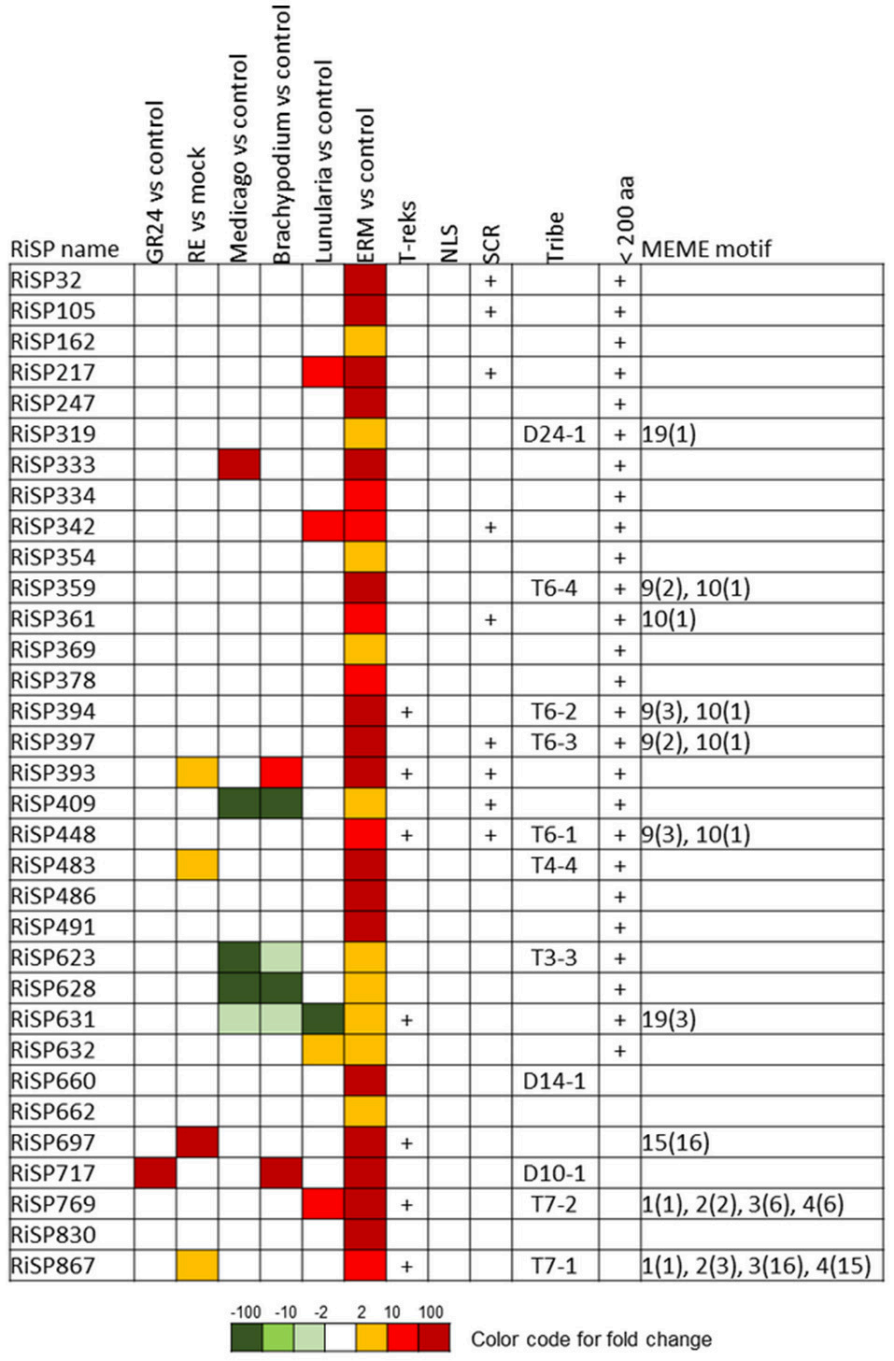
**RiSPs of unknown function preferentially up-regulated in ERM**. MEME motifs are described by their number (Figure 4 and Figure S2) and their occurrence (value within brackets).

**Figure 7:**
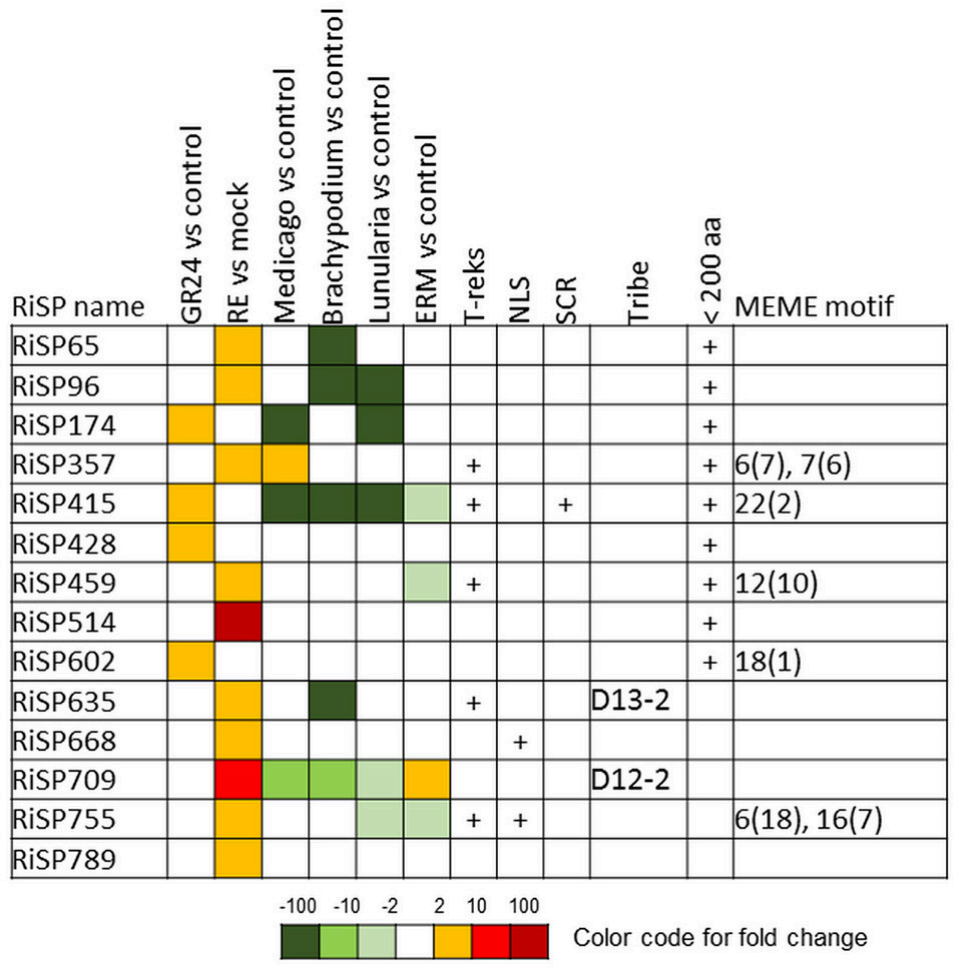
**RiSPs of unknown function preferentially up-regulated after perception of plant signals**. MEME motifs are described by their number (Figure 4 and Figure S2) and their occurrence (value within brackets).

688 among 872 RiSPs lack PFAM annotation and have no predicted function. We used the MEME software to identify motifs present as internal repeats or shared by different proteins that may unravel functional groups not yet described (Figure 4; Figure S2), before sorting them by expression groups. Within RiSPs with no PFAM domain, we identified 44, 33, and 14 RiSPs with a preferential expression pattern in IRM, ERM or in response to plant signals, respectively (Figures 5–**7**). In RiSPs preferentially expressed in IRM (Figure 5), 27 genes show a specific up-regulation in all three hosts tested, whereas six are also induced in response to plant signals and six also induced in ERM. Among these 27 RiSPs, three display a motif containing a RXLX sequence and are not induced in the ERM (motif 18, Figure 4; RiSP522, RiSP535, and RiSP546; Figure S2). The RXLX motif is reminiscent of RXLR effectors found in oomycetes (see next section).

### Search for candidate effectors

Effectors are SPs that have an incidence on host cell metabolism or immunity. Although functional validations are necessary to identify SP coding genes, *in silico* analysis allows the prediction of candidate effector proteins. Genes coding for effector proteins are often up-regulated during early and/or late stage of interaction with the host. Effectors of many different sizes have been reported in the litterature dedicated to bacteria, oomycetes, or fungi. Therefore, we did not apply a size criterion and SPs of all sizes can be assigned to the effector category. However, since fungal effectors are usually relatively small (< 200 aa), we report this additional protein feature in our description. Finally, fungal effectors often do not possess any known functions and may present structural features such as a NLS for nuclear host cell targeting, a high number of Cysteine for increasing protein stability in plant apoplasm (Ellis et al., 2009), or internal repeats such as SP7 and Ss-RhS1 (Kloppholz et al., 2011; Yu et al., 2016).

A total of 41 SP-coding genes containing a NLS motif were identified from the 872 RiSP candidates (Table S1C). Only four are up-regulated. RiSP668 and RiSP755 (**Figure 7**) are up-regulated in reponse to root exudates, RiSP646 (Figure 5) in IRM and RiSP734 in both conditions (Figure 5). The software MEME did not identify any significant enriched putative translocation motif in these four proteins or in the 41 NLS-containing RiSPs.

Throughout the 872 RiSPs, 141 can be assigned to Small Cysteine Rich (SCR) proteins. The analysis of the 872 RiSPs did not reveal an enrichment of Cysteine-containing motifs compared to the whole genome repertoire. In order to identify the motifs conserved in SCRs, we run the MEME software in this subgroup of proteins but no motif enrichment was observed. Similarly, no enrichment was detected in 127 SCRs predicted to form disulfide bond by Disulfind. Although *R. irregularis* produces SCRs, they did not form expression clusters in our conditions, as it was already observed for fungal pathogens (Saunders et al., 2012).

In addition to SCRs, Repeat Containing Proteins (RCPs) are often found in the secretome of eukaryotic filamentous plant pathogens (Mueller et al., 2008). The identification of RCPs was performed in all RiSPs using intra-sequence investigation (T-reks algorithm—Jorda and Kajava, 2009) (column D in Table S1C, see methods). On the 110 up-regulated genes with no PFAM domain, the motif discovery tool MEME (Bailey et al., 2009) was then used to identify motifs present in different proteins to reveal functional groups of proteins sharing domains yet uncharacterized (Figure 4; Figure S2). An intra-sequence MEME search allowed a visualization of the repeated motifs previously identified by the T-reks algorithm (Figure 4; Figure S2). As observed in the basidiomycete fungus *Ustilago maydis*, we identified two classes of RCPs where the repeated motifs display or not a putative KEX2 protease cleavage site, which consist of a dipeptide containing a Lysine or an Arginine followed by an Arginine: [KR]R (Mueller et al., 2008). In plant-fungus interactions, limited information is available for such proteins predicted to be cleaved and secreted as peptides. In the RCPs predicted to be cleaved, a first group is mainly up-regulated in ERM: RiSP752, RiSP769 and RiSP867 (Figures 4, 6). These three RCPs have striking similarities with SP7, a previously characterized effector of *R. irregularis* (Kloppholz et al., 2011; Lin et al., 2014). These four RiSPs share the motifs 2 and 3. An alignment of SP7 with these RiSPs reveals indeed key conserved amino acids within the repeated motifs and a very strong conservation of the predicted KEX2 cleavage site (Figure S3). These observations suggest that SP7 and its relatives may be cleaved into short peptides. A second group of RiSPs putatively cleaved is mostly expressed *in planta* and present repetitions of the very short motifs 6, 7, and 8. Finally, four RiSPs with up-regulation in ERM display the motif 9 in two or three copies and is followed by the C terminal motif 10. This last motif is also found alone in RiSP361.

RCPs without cleavage sites were proposed to play a role in fungal cell wall as repetitive proteins (Mueller et al., 2008). Ss-Rhs1, a *Sclerotinia sclerotiorum* SP, was also recently reported as important for virulence (Yu et al., 2016). In *R. irregularis*, seven repetitive proteins were identified (Figure 4; Figure S2, motif 11 to 17). Our expression analysis reveals that these proteins are expressed at different physiological stages: RiSP604 and RiSP718 are IRM specific (Figure 5), RiSP459 and RiSP755 are up-regulated in response to root exudates (Figure 7), RiSP612 and RiSP735 are induced in both IRM and ERM (Figure 5) and RiSP697 is induced in ERM and in response to plant exudates (Figure 6). The motif 19, a Histidine rich motif, is present in four proteins (Figure 4; Figure S2). The motif 18, that contains a RXLX sequence, that resembles the well-characterized RXLR motif in effectors of oomycetes (Whisson et al., 2007), is present in four proteins, either strongly expressed *in planta* (RiSP522, RiSP535, RiSP546, Figure 5) or induced in response to GR24 treatment (RiSP602, Figure 7).

We then searched for all known motifs identified in effectors of eukaryotic filamentous plant pathogens: [LI]xAR, [RK]Cx_2_Cx_12_H (Yoshida et al., 2009), RxLx (Plett et al., 2011), RxLR, [YFW]xC (Godfrey et al., 2010), YxSL[RK] (Lévesque et al., 2010), [WYF]CxTYxSTYL, [SG]PC[KR]P (Sperschneider et al., 2013), G[IFY][ALST]R (Catanzariti et al., 2006), CHxC (Kemen et al., 2011), [FY][MR][HY]V[AE]Y[PR]CM, CL[AK][TW]LHM, [WI][HG]N[WE] (Louis et al., 2014), KECxD (Nicastro et al., 2009), RSIDELD (Zuccaro et al., 2011), IGYRxVxxxxA, K[AV]W[VI]P, Q[ML]LIP (Cheng et al., 2014). Most of these motifs were present in *R. irregularis* secretome, however none of them was significantly enriched compared to non-secreted proteins (Table S4). Furthermore, we were unable to identify a positional constraint for these motifs. Previously, Lin and collaborators interestingly noticed that *R. irregularis* presents CRN-type effectors (Lin et al., 2014). These effector proteins are also present in the chytrid fungus *Batrachochytrium dendrobatidis* (Joneson et al., 2011). Three CRN-like proteins are present in the 872 SPs (RiSP187, RiSP488, and RiSP756), but none of them present an expression pattern of interest (Tables S1B,C). One is expressed at a low basal level in all conditions tested (RiSP187), the two others have no or very weak expression levels in our experimental conditions (RiSP756 and RiSP488).

Finally, all genes coding for proteins with unknown function and up-regulated either specifically in one host, or up-regulated in all hosts tested (Figure 2 and Table S1) are candidates to fulfill an effector role, even though they do not display structural features of interest: NLS, *de novo* identified motif, repeated motifs or Cys-rich content. It is for example worth mentioning RiSP646 that displays a NLS, is upregulated in all three tested hosts and is conserved in *P. indica, T. melanosporum* and *L. bicolor*. This SPs may be a good candidate effector acting in a large range of mutualistic fungi.

### *Gigaspora rosea* secreted proteins up-regulated during symbiosis

Previous RNA sequencing data were obtained from *G. rosea* cultivated in the same conditions as for *R. irregularis*, except that we were unable to obtain mycorrhized thalli of *Lunularia* (Figure S1). The *G. rosea* transcriptome assembly consists of 86332 contigs (mean length: 643 bp; 13318 contigs > 1 kb, 57472 contigs > 300 bp) (Tang et al., 2016). Similarly to *R. irregularis*, we curated *G. rosea* secretome for the presence of NLS, PFAM domains, SCR and T-reks features and used Blastclust to identify protein tribes. We obtained 2633 GrSPs (Table S5C), a number that could be however overestimated as defined from a transcriptome assembly of 86332 non-redundant virtual transcripts that contain probable partial sequences (Tang et al., 2016). We displayed in Table S5 the GrSPs that present homologies with *G. margarita* SPs, although this comparison is limited by the use of a minimal cut-off size of 350 bp in this assembly (Salvioli et al., 2015). Few paralog groups of proteins were identified among GrSPs using Blastclust: Only 87 proteins were grouped in 20 tribes of at least 3 members. Seventy-eight other proteins were present in tribes of two members. A total of 42, 78, and 625 GrSPs presented respectively NLS, T-reks and SCR features while 152 displayed a PFAM domain, including 16 CAZymes. Only five motifs were identified by the MEME discovery tool in GrSPs, either as repeated motifs or shared by different proteins (Figure S4). The motif “a” presents a putative KEX2 cleavage site.

**Figure 8:**
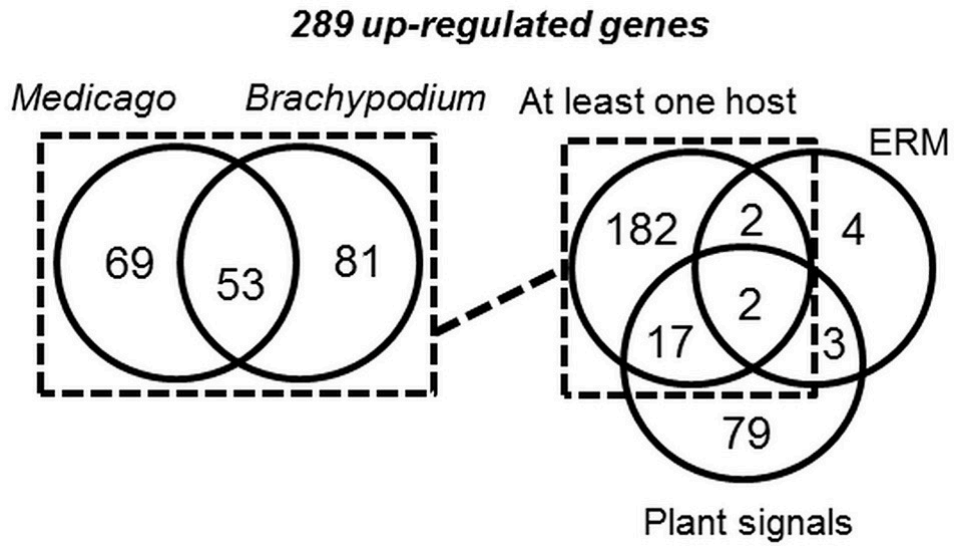
**Venn diagram of *G. rosea* genes up-regulated in the different comparisons**. Genes up-regulated in at least one host (left panel), were pooled and compared to genes up-regulated in ERM or in germinating spores treated with plant signals (GR24 or plant exudates) (right panel).

When analyzing expression patterns, only 289 GrSPs are found statistically significantly up-regulated in at least one tested condition (Figure 8 and Table S5D). Nine of them encode proteins with a NLS, 77 are assigned to SCRs, 13 display internal repeats and 40 present a PFAM domain (Figure S5). Eleven of them code for proteases including seven Aspartic protease already highly abundant in *R. irregularis* up-regulated genes. Proteins with domains involved in protein-protein interaction (Kelch, TPR) or known as chaperone (DnaJ, not previously described as secreted proteins) are also present. Additionally, proteins potentially involved in fungal/host cell wall modification were found: One polysaccharide deacetylase, two glycosyl hydrolases, one glycosyl transferase and one GDSL lipase-like. We already described proteins involved in cell wall modification in *R. irregularis*, but these proteins displayed different domains from those of *G. rosea*.

Considering the 289 up-regulated GrSP genes (Table S5D; Figure 8 right panel), a majority (203) is up-regulated *in planta*, among which 53 are commonly up-regulated in Medicago and Brachypodium (Table S5E). In this set, 29 are short proteins (< 200 aa) including 9 SCRs. Seventeen out of the 53 common GrSPs present a PFAM including six proteases. Two others (GrSP2592 and GrSP2603) are predicted to possess both a PFAM domain (a Calcineurin-like phosphoesterase and DnaJ chaperone respectively) and a NLS, suggesting a regulatory function in the host nucleus.

The other up-regulated GrSPs *in planta* are host-specific: 150 among 203 GrSPs, with 69 and 81 GrSPs respectively specific to Medicago and Brachypodium (Figure 8; Table S5F). Most of them (134) correspond to short proteins (< 200 aa), 46 are SCRs and three contain a NLS (GrSP711, GrSP1384, and GrSP1700). Accordingly to these investigations, *G. rosea* displays a larger proportion of host specific SPs (74%) than *R. irregularis* (44%).

### Definition of a core set of SPs shared by *R. irregularis* and *G. rosea*

**Figure 9:**
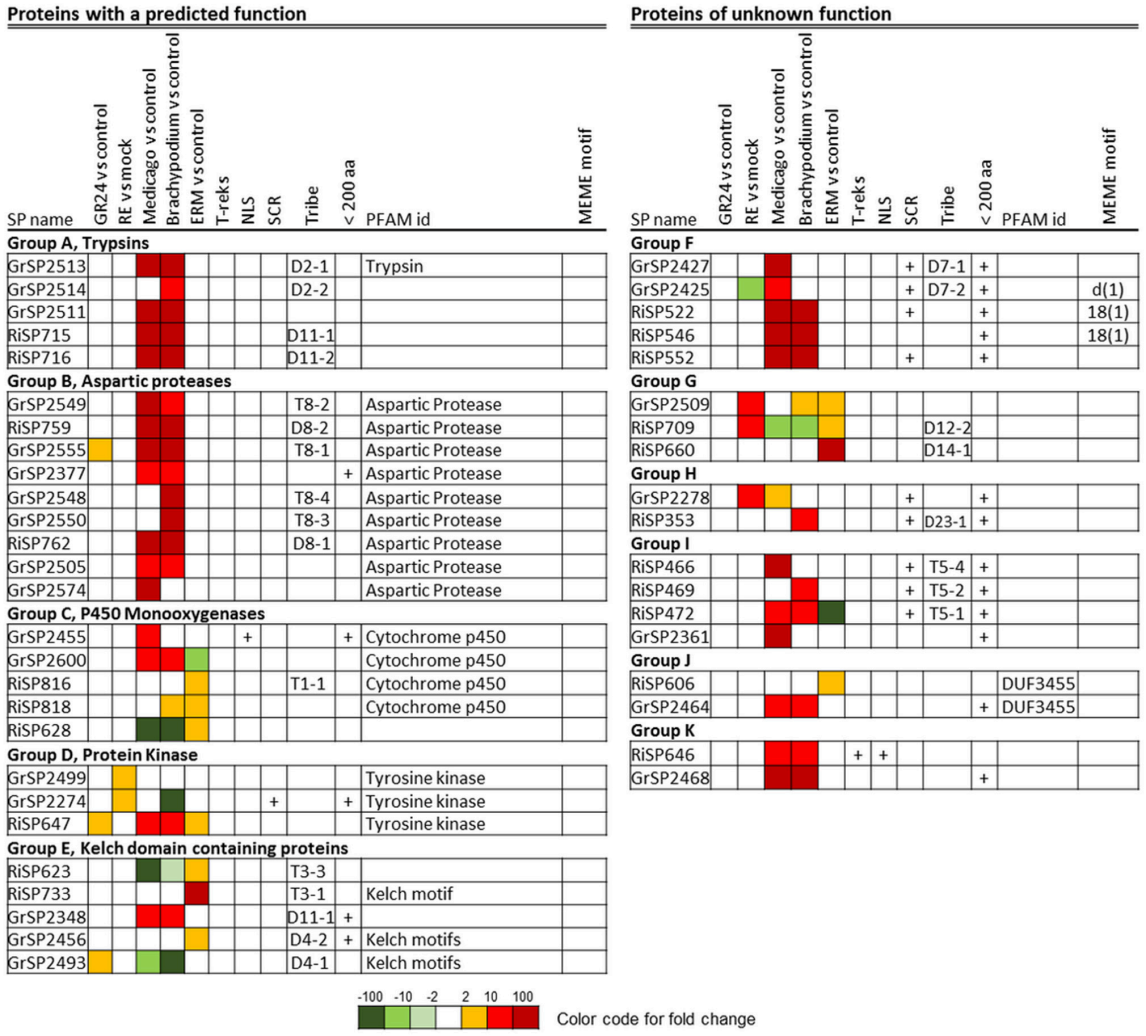
**Core up-regulated secretome in *G. rosea* and *R. irregularis***. Up-regulated RiSPs and GrSPs were compared through a reciprocal blast analysis. Proteins showing a similarity with at least one protein in the other fungus (e-value < 10^−5^) were retained and sorted by sequence groups.

A BlastP analysis revealed that 194 out of 872 RiSPs (22%) present sequence similarities with GrSPs even with an e-value lower than 10^−1^ (Table S1C column AB). In order to identify a possible conserved core secretome expressed at the different steps of the symbiosis, we considered RiSPs and GrSPs that showed homology through a blastP analysis at an e-value < 10^−5^. It resulted in 21 RiSPs and 24 GrSPs, sorted in 11 sequence groups (Figure 9). Within PFAM domain-containing proteins, we identified several Aspartic proteases and trypsins, but also proteins containing a Kelch domain, involved in protein-protein interaction. Kinases and Cytochrome P450 were also present. Interestingly, some proteins of unknown function clustered with proteins containing a PFAM domain, thus suggesting a putative similar function. For example in group A (Figure 9), three GrSPs belonged to the same group even though only one of them contains a Trypsin domain. They were grouped with two RiSPs for which a predicted trypsin-like activity was also infered. In groups F to K, secreted proteins with unknown functions were present, including the above mentionned RiSPs with a RXLX containing motif (group F). The RXLX motif is however not conserved in GrSPs of the same orthologous group.

These 45 genes did not always display the same expression patterns in *G. rosea* and *R. irregularis* (Figure 9). For example in group D (tyrosine kinase), RiSP647 was found up-regulated *in planta*, in ERM and in response to plant signals while the GrSP2274 and GrSP2499 orthologs were exclusively up-regulated in response to root exudates. In group C, P450-coding GrSPs were induced *in planta* whereas in *R. irregularis* they were mostly up-regulated in ERM. The DUF3455 containing proteins in *R. irregularis* and *G. rosea* also displayed striking difference in expression (group J). Within the 11 groups, six are formed by SPs that have conserved expression pattern in *G. rosea* and *R. irregularis*: Group A and B that contain proteases quite exclusively expressed *in planta*; groups F, I, and K, that are composed of SPs of unknown function up-regulated *in planta*; and group G, formed by SPs of unknown function mainly up-regulated by plant signals and in ERMs. These groups form the core set of SPs shared by *R. irregularis* and *G. rosea* that could have conserved roles during the establishment of AM symbiosis. Throughout the SPs presented in Figure 9, a smal fraction shows conservation in other beneficial fungi (groups B, C, D, and K), thus suggesting a broader role in plant/mutualistic fungi interactions.

## Discussion

Secreted proteins (SPs) are major actors of fungal cell organization and development such as cell wall structure (e.g., chitin deacetylase, structural proteins), mating, and nutrient acquisition (e.g., hydrolases) (Thorner, 1981; Bouws et al., 2008). SPs are also described for their role in host invasion as some of them can be effectors that are key outposts modulating or altering host immunity (Koeck et al., 2011; Petre et al., 2014; Plett and Martin, 2015). In this work, we first defined sets of secreted protein (SP) genes from two phylogenetically distant AM fungi—*R. irregularis* and *G. rosea*, based on gene model definition and gene expression activity. We then compared the expression patterns of SPs during the establishement of AM symbiosis in the two AM fungi associated with different host plants in order to investigate the conservation of their invasion strategies. Our interest particularly focused on the following questions: (i) Does *R. irregularis* use the same set of SPs whatever the host plants or are there host-specific SPs in *R. irregularis*? (ii) Are expressed secretomes of *R. irregularis* and *G. rosea* reflecting similar strategy for plant invasion? (iii) Are there “universal keys” for AM fungi to invade diverse host plants?

### Secretome sets of *R. irregularis* and *G. rosea*

We identified 872 RiSPs representing 2.4% of the 35893 predicted proteins present in the proteome of *R. irregularis*. It is interesting to compare this repertoire to those of other plant interacting fungi, like the maize pathogen *U. maydis* (431 predicted SPs, 6.6% of the proteome) (Lum and Min, 2011), the rice pathogen *Magnaporthe oryzae* (1471 predicted SPs, 10.5% of the proteome) or the multi-host ectomycorrhizal fungus *Laccaria bicolor* (650 predicted SPs, 3.6% of the proteome - Lum and Min, 2011). Considering that *R. irregularis* can interact with thousands of host plants, one could have expected a much larger set of putative effector proteins in the secretome, different ones for different hosts. However, it has been documented that fungal secretome size is the result of environmental and evolutive adaptive traits as various as life-style, host spectrum, genome, and proteome sizes (Meinken et al., 2014; Pellegrin et al., 2015; Kim et al., 2016). Since the number of RiSPs is not higher than average, we can speculate that this fungus evolved with a limited number of effectors to possibly interact with conserved targets. In the case of *G. rosea*, a larger set was identified from the transcriptome assembly (2633 GrSPs, i.e., 2.7% of the gene repertoire), although it can be estimated that genomic data would resolve some isoforms and fragmented ORFs. When the *G. rosea* genome will be available, these results will be enhanced and fine-tuned. When comparing these GrSPs with RiSPs (this study), we found that only 22% have sequence similarities, whereas 95% of RiSPs have similarities with SPs defined from *Rhizophagus clarus* (Sędzielewska Toro and Brachmann, 2016). This result indicates that SPs are mainly composed of lineage specific proteins. This is in agreement with previous findings in litterature on interspecies comparative analysis of the secretomes. Closely related fungal species, either parasitic (Schirawski et al., 2010; Heard et al., 2015) mutualistic or saprotrophic (Pellegrin et al., 2015), obvioulsy present highly conserved secretome or effectome. Different factors can contribute to secretome variation and evolution: Host specificity (Dutheil et al., 2016), phylogenetic history (Krijger et al., 2014; Pellegrin et al., 2015) and also life-style (Kim et al., 2016). *G. rosea* and *R. irregularis* sharing the same lifestyle, the great divergence of their secretome should mostly result from differences in host range and phylogenetic history of these two species.

### Search for putative effectors

The first effector described in AMF was SP7 in *R. irregularis* (Kloppholz et al., 2011). Interestingly, and in agreement with previous findings, three RiSPs were found to have similarities with SP7 (Lin et al., 2014). These RiSPs were found up-regulated *in planta* and also highly up-regulated in ERM. Our sequence analysis revealed that these genes contain repetitive motifs all starting with a conserved ([KR]R) signature previously identified as a KEX2 proteolytic cleavage site. An example of KEX2-cleaved proteins is Rep1 from *U. maydis* that produces 11 secreted peptides involved in cellular attachment (Teertstra et al., 2009). SP7 was described to act as a native protein, repressing the expression of a plant transcription factor in the nucleus. The presence of KEX2-cleavage sites suggests that SP7 and its orthologs might also be present in the fungal/plant interface as short peptides previously maturated in the fungal Golgi. Two proteins in the *G. rosea* proteome also present repeated motifs containing a putative KEX2-cleavage site (GrSP2479 and GrSP2488). However, their sequence is different from that of the *R. irregularis* “SP7 family.” Further functional characterization will unravel whether these proteins are indeed processed and secreted as short peptides and how these peptides act, either as cell wall attached components similarly to Rep1 or whether they fulfill a different role on the plant surface or within plant cells.

Another putative effector, SIS1, was recently published (Tsuzuki et al., 2016). This gene (corresponding to the Gloin1 transcript ID 342269) was not included in our analysis since a transmembrane domain was detected by our bioinformatic pipeline.

Among the different RiSPs that we analyzed, none have strong similarities with known fungal virulence proteins (not shown). We attempted to identify large groups of gene families likely encoding effector proteins, as it was found in oomycetes species. Oomycetes display large expanded genes families containing conserved N-terminal motifs, proposed to be crucial for translocation into plant host cells and for virulence (de Jonge et al., 2011; Giraldo and Valent, 2013). Within these gene families, the encoded RXLR and CRN effectors are the most well-studied. In fungi, there is no functional evidence of expanded gene families sharing large conserved domains. However, a conserved motif (Y/F/WxC), proximal to the signal peptide, was observed in a large proportion of secreted proteins in *Blumeria* and *Puccinia* species (Godfrey et al., 2010). Four RXLX-motif-containing proteins were identified in the RiSP set. While in oomycetes a RXLR sequence is proximal to the signal peptide, we only found the RXLX motif (18) in central position of *R. irregularis* proteins. It remains to be determined whether these proteins are true effectors and if this RXLX motif is important for protein translocation into host cells, as it is the case for miSSP7 in *L. bicolor* that contains a RXLX motif (RALG sequence, Plett et al., 2011) required for host cell entry. Interestingly, three of the SPs that contain this motif were specifically expressed *in planta* whatever the hosts tested. Conversely, while some RiSPs contain a CRN-like motif, they do not display an expression profile suggesting a role as effector. In the case of GrSPs, the typical CRN amino acid sequence LFLAK (Haas et al., 2009) was not found, and *de novo* search for enriched motifs did not allow identifying sequences containing a RXLX motif.

Another feature frequently observed in the secretome of eukaryotic filamentous plant pathogens is the presence of families of small cysteine rich proteins. The RiSP set includes 141 SCR proteins, and 625 for the GRSP set. In the two sets, SCR proteins were not organized in protein families as defined by Blastclust. Different SCR proteins from *R. irregularis* and *G. rosea* were highly induced in at least one host (24 and 55 respectively), suggesting a role at the plant-fungus interface.

Even though host cell wall degrading enzymes are absent in *R. irregularis* (Tisserant et al., 2013) and *G. rosea* secretomes (Tang et al., 2016), we identified proteins that may act as plant cell wall modifiers. Lignin targeting enzymes were present such as a laccase-like (RiSP847) induced in the three hosts and a glyoxal oxidase domain-containing protein (RiSP833) up-regulated only in *B. distachyon* and in ERM. Many enzymes acting as glycoside modifiers were also identified in GrSPs (e.g., the glycoside hydrolases GrSP2566 and GrSP2573, or GrSP2525, a polysaccharide deacetylase). A CAP-domain-containing protein (RiSP574) may also be involved in cell wall remodeling or cell-cell adhesion. This protein is induced in ERM, as observed for the group of proteins with lipid binding motifs (ML motifs). Altogether, they may play an important role in the sensing machinery devoted to host recognition. Since proteins with ML domains are also present in fungal pathogen secretomes (Saunders et al., 2012), one could also consider that they may be involved in targeting specific host membrane regions, possibly involved in PAMP perception.

Through our *de novo* searching for unknown motifs, we identified as His-rich motif in two genes expressed specifically in ERM (RiSP319 and RiSP631) and in two genes expressed *in planta* (RiSP531 and RiSP256). Interestingly, these motifs have been proposed to have a metal binding property and are present in plant cell wall arabinoglactan proteins (Hijazi et al., 2014) and in plant dehydrins, where they regulate plasma membrane stability (Hara et al., 2005). Based on this observation, an effector role can be also suspected for these four proteins, acting at the plant-fungus interface.

Lastly, it is interesting to point out that numerous aspartic proteases and trypsins present in RiSP and GrSP sets are up-regulated *in planta* in these two species. They are good candidates in the targeting of plant defense proteins such as chitinases.

### Comparison of RiSP and GrSP expression patterns in different hosts: different AM fungi, different invasive strategies?

Comparison of RiSPs gene expression patterns in different hosts revealed that an important proportion of them is shared whatever the plant host. Even in *L. cruciata*, where the symbiosis occurs in a photosynthetic thallus and not in root tissues, the expression pattern presented a strong consensus with those obtained from roots of *Brachypodium cruciata* and *M. truncatula*. Due to the large phylogenetic diversity of these hosts, and the physiological difference in the colonized tissues, we propose that this set of genes represents the common mycorrhizal RiSP set. In addition to this common set of genes, *R. irregularis* encodes other RiSPs specifically expressed in the different hosts tested. As AM symbiosis is a continuous process (young and old colonization structures are concomitant in one root), it is more likely that these differences are due to specific expression in one host than an absence of expression due to different stage of development in another host. We speculate that these RiSPs may be specific effectors that could have a role in the fitness to certain hosts. Interestingly, we observed that these “host specific SPs” are mostly of small size and contain no PFAM domain. The three plant host species investigated in this study belong to highly phylogenetically divergent clades, so it can be proposed that different combinations of these “host specific” RiSPs would be expressed according to different hosts, in addition to the commonly expressed RiSP genes.

In *G. rosea*, a larger ratio of “host specific” GrSPs was found. When only looking at *M. truncatula* and *B. distachyon* IRM data, we observed that 74% of up-regulated *in planta* GrSPs are expressed in only one host, compared to 44% for RiSPs. “Host specific” GrSPs do not have orthologs among RiSPs and reciprocally, supporting the lineage specific origin of the “host specific” SPs. Genome size, gene repertoire and secreted gene set of *G. rosea* are larger than those of *R. irregularis*. This gene inflation might be correlated to an increase of “host specific” GrSPs. However, this increase of putative host specific GrSPs is not linked to a higher efficiency of symbiotic ability as *G. rosea* and Gigasporaceae in general, are described as less aggressive than *R. irregularis* and Glomales (Jansa et al., 2002; Russell and Bulman, 2005; Sýkorová et al., 2007). As an illustration, we failed to obtain mycorrhizal association of *G. rosea* with *L. cruciata* despite numerous attempts, in accordance to diversity analyses and colonization assays performed on other liverworts (Ligrone et al., 2007). In the same line, mycorrhizal association of *G. rosea* DAOM 194757 with *M. truncatula* A17 is slower than with *R. irregularis* DAOM 197198 so that we used a nursery system in the 3 first weeks of interaction to obtain a high level of symbiotic structures in 5 weeks as for *R. irregularis*. The contrast between the symbiotic developmental strategies of these two fungi, coupled to our data on the numerous host-specific GrSPs, argue for a higher degree of “host perference” of *G. rosea* as observed for some pathogens (Poloni and Schirawski, 2015).

### Identification of a set of common SPs between *R. irregularis* and *G. rosea*

We identified several SP-coding genes that share similarities of sequence and expression pattern in different hosts, several of them being only expressed in host tissues. They code for proteases and SP of unknown function and represent a highly conserved AM symbiotic core secretome, predicted to play a conserved and presumably essential role for the establishement of AM symbiosis. This low number of highy conserved SPs was however expected since *R. irregularis* and *G. rosea* are phylogenetically distant AM fungi. Nevertheless, discovering the function of these SPs will definitely unravel key conserved molecular mechanisms that may be universal to an extremely large number of AMF—plant interactions. These SPs may also play a role beyond AMF, since some of them are conserved in other mutalistic fungi: The endophyte *P. indica* or the ectomycorhizal fungi *L. bicolor* and *T. melanosporum*. In addition to their conserved SPs, *R. irregularis* and *G. rosea* secretomes share numerous common features. Both fungal species lack canonical cell wall degrading enzyme, protease inhibitors or known fungal effectors, suggesting that they colonize host roots by mechanisms far different from root pathogens. These two secretomes include an important set of diverse enzymes like P450 monooxygenases, aspartic and serine proteases. These last ones can act as factors of compatibility by targeting host chitinases, as proteases of plant fungal pathogens were described as virulence factors (reviewed by Jashni et al., 2015). They can also play a role in signaling pathways via activation or inactivation of host/fungal proteins through specific cleavage, thus turning on a pathway leading to root colonization. Besides, they may also fulfill a role in amino acid acquisition after cleavage of extracellular proteins or peptides. We also identified SPs both in *R. irregularis* and *G. rosea* that may target fungal or plant cell wall during the symbiosis, even though these proteins present very different PFAM domains between the two fungi. This observation suggests that same targets may be shared by the two fungi, even though the nature of the involved SPs are different.

To conclude, our study comparing SP gene sets and their expression from two phylogenetically distant AM fungi in different hosts shed light at the molecular level on the existence of different symbiotic strategies among glomeromycotan fungi (Smith et al., 2003). First, the ubiquist species *R. irregularis* harbors an important core of symbiotic SPs whatever the host, corresponding to a lower host specialization than *G. rosea*. Secondly, SPs expressed while colonizing host plants were mostly fungal lineage specific. Third, a core set of symbiotic SPs shared by *R. irregularis* and *G. rosea* was identified, including putative effector proteins that could target conserved mechanisms in monocots and dicots. This work therefore paves the way to further functional analysis dedicated to proteins specifically or commonly expressed by different glomeromycotan fungi, underlying that data obtained from *R. irregularis* DAOM 197198 could not always be relevant for all AM fungi as often considered in litterature.

## Author contributions

CR and NFdF designed the experiments. NT and MM respectively realized the experiments with *G. rosea*. and *R. irregularis*. LK, NT, MM, and CR. analyzed the RNAseq data. HS developed the scripts for the *in silico* analyses. LK, MLM, CR, and NFdF analyzed and interpreted the secretome data. LK, CR, and NFdF wrote the manuscript.

### Conflict of interest statement

The authors declare that the research was conducted in the absence of any commercial or financial relationships that could be construed as a potential conflict of interest.

## Supplementary material

Figure S1 **Overall presentation of the different biological conditions prepared for RNAseq comparisons**. *G. rosea* and *R. irregularis* were both cultivated alone or in symbiosis with different hosts. Germinated spores *in vitro* (asymbiotic stage) were used as reference for all comparisons. See material and methods for details. FC, fold change.

Figure S2 **List of the motifs identified by the MEME discovery tool and presented in Figure 4**. Boxes indicate putative KEX2 clivage sites ([KR]R). A non-canonical clivage site is underlined in Motif 9.

Figure S3 **Sequence alignment of SP7 with three RiSPs displaying strong similarity**. Alignment was made with MAFFT (v7.299b) and displayed with CLUSTAL format. Signal peptide was removed from the protein sequences. Boxes indicate putative clivage sites ([KR]R) followed by conserved amino acids.

Figure S4 **Protein structures (A) of GrSPs displaying motifs of unknown function (B)**. A putative KEX2 cleavage site is underlined in motif ≪ a ≫.

Figure S5 **GrSPs with a PFAM domain up-regulated in at least one comparison**. RE, root exudates; ERM, Extra-Radical Mycelium; NLS, Nuclear Localization Signal; SCR, Small Cysteine Rich.

Table S1 **Sequences, structural features, and expression of *R. irregularis* gene-encoding secreted proteins. (A)** Overall quality of RNAseq libraries from *R. irregularis* in culture or in symbiotic tissues. Triplicate variability (figure) was analyzed by principal component analysis with CLC Genomic Workbench. For each replicates, the correlation matrix (“normalized” version of the covariance matrix) was calculated to define the orthogonal eigenvectors of the first and second major principal components, showing a simplified version of the variability of data. **(B)** RNAseq raw expression data of the 872 RiSPs obtained from Illumina Hiseq2000 sequencing (2 x 101 bp). Calculations were performed using CLC Genomics Workbench 9.0 software (see methods). Control = *in vitro* germinated spores (except for root exudates assays: control = spores produced in same condition but without root exudates). **(C)** List of the 872 RiSPs, their structural features and expression level in different comparisons. Column A refers to the best gene models that were identified by comparing Gloin1 and Rir gene models by GMAP. GeneID listed in Column B refers to the non-selected SP sequences (if present in the other assembly) that have similarity with the best SP gene model in column A; GeneID in bold type in column B refer to sequences that are strictly similar (identity and length) to corresponding sequence in Column A. RiSP name in column C corresponds to the nomenclature used in the present manuscript. Columns D to R list the sequence features. MEME motifs are described by their number (Figure 4) and their occurrence (value within brackets). Expression data are listed in column T to Y. Only statistically validated fold change values (FDR ≤ 0.05) are presented (see S1B for global expression data). Best orthologs with sequence of *R. clarus* are listed in columns Z and AA, and with sequences of *G. rosea* in column AB. Gene id for *L. bicolor, T. melanosporum*, and *P. indica* are annotated with their JGI gene number, preceded by “Lb,” “Tm,” and “Pi” respectively. **(D)** List, expression and structural features of the 33 RiSPs overexpressed in the three tested hosts. Only statistically robust data are listed (fold discovery rate—FDR ≤ 0.05). **(E)** List, expression, and structural features of the 28 RiSPs up-regulated in a single host. Only statistically robust data (fold discovery rate—FDR ≤ 0.05) are listed. RE, Root Exudates; ERM, Extra-Radical Mycelium; FDR, False Discovery Rate; NLS, Nuclear Localization Signal; PFAM, Protein Family; SCR, Small Cysteine Rich; SP, Signal Peptide. **(F)** Expression level of 10 RiSPs genes in mycorrhized *M. truncatula* roots compared to germinating spores of *R. irregularis*, as defined by RNAseq and RT-qPCR. RNA was extracted with the Qiagen RNAeasy mini kit and cDNA was synthesized using the Promega M-MLV RT enzyme, following the manufacturer instructions. RT-qPCR were performed on a Roche LightCycler 480. Total RNA were extracted from mycorrhized *M. truncatula* roots and germinating spores, in three independant replicates each. Average of the three experiments is depicted. Gene expression were normalized using *R. irregularis* PFK gene expression by the 2-ΔΔCT method.

Table S2 **Enriched PFAM domains in *R. irregularis* secretome**. Domains present in at least 3 proteins are depicted. Enrichment was determined through frequence comparison against the whole proteome. *P*-value calculation for statistical enrichment was calculated with a Chi-square test and a Bonferroni correction.

Table S3 **PFAM domains enriched in up-regulated RiSPs**. Only PFAM domains present in at least 3 proteins were selected. Enrichment was calculated against the whole proteome. *P*-value calculation for statistical enrichment was calculated with a Chi-square test and a Bonferroni correction.

Table S4 **Known effector motifs search through *R. irregularis* secretome**. Occurrence of the different motifs is indicated, after allowing 0 or 1 amino acid mismatch. *P*-value calculation for statistical enrichment against non-secreted proteins was calculated with a Chi-square test and a Bonferroni correction. Reference for each motif is indicated in the text.

Table S5 **Sequences, structural features, and expression of *G. rosea* gene-encoding secreted proteins. (A)** Overall quality of RNAseq libraires from *G. rosea* in culture or in symbiotic tissues. Triplicate variability (figure) was analyzed by principal component analysis by using CLC Genomic Workbench. For each replicates, the correlation matrix (“normalized” version of the covariance matrix) was calculated to define the orthogonal eigenvectors of the first and second major principal components, showing a simplified version of the variability of data. **(B)** RNAseq raw expression data of the 2633 GrSPs obtained from Illumina Hiseq2000 sequencing (2 x 101 bp) and Miseq1000 sequencing (2 x 151 bp). Calculations were performed using CLC Genomics Workbench 9.0 software (Control = *in vitro* germinated spores, except root exudate assays: mock = spores produced in same condition but without root exudates). **(C)** List of the 2633 GrSPs, structural features and expression level in different comparisons. “Gr id” refers to number of contig as defined in Tang et al. (2016). Columns C to O list the sequence features. MEME motifs are described by their number (Figure 4) and their occurrence (value within brackets). Expression data are listed in column P to T. Only fold change values statistically validated (fold discovery rate—FDR < 0.05) are presented (see Table S5B for global expression data). Best orthologs with sequence of RiSPs are listed in column V. Putative isoforms are depicted with superscripts number in “Gr id” column, see footnotes. **(D)** List, expression, and structural features of the 289 GrSPs up-regulated in at least one symbiotic condition. Only statistically robust data (FDR ≤ 0.05) are listed. **(E)** List, expression, and structural features of the 53 GrSPs up-regulated in the two tested hosts. Only statistically robust data (FDR ≤ 0.05) are listed. **(F)** List, expression, and structural features of the 150 GrSPs up-regulated in single host. Only statistically robust data (FDR ≤ 0.05) are listed. RE, Root Exudates; ERM, Extra-Radical Mycelium; FDR, False Discovery Rate; NLS, Nuclear Localization Signal; PFAM, Protein Family; SCR, Small Cysteine Rich; SP: Signal Peptide.
