# Corrigendum: The Comparison of Expressed Candidate Secreted Proteins from Two Arbuscular Mycorrhizal Fungi Unravels Common and Specific Molecular Tools to Invade Different Host Plants

**DOI:** 10.3389/fpls.2017.02065

**Published:** 2017-12-08

**Authors:** Laurent Kamel, Nianwu Tang, Mathilde Malbreil, Hélène San Clemente, Morgane Le Marquer, Christophe Roux, Nicolas Frei dit Frey

**Affiliations:** ^1^Laboratoire de Recherche en Sciences Végétales, Université Paul Sabatier - Université de Toulouse, Centre National de la Recherche Scientifique, Castanet-Tolosan, France; ^2^Agronutrition, Laboratoire de Biotechnologies, Labege, France

**Keywords:** Glomeromycota, secretome, comparative transcriptomics, effectors, symbiosis

In the original article, there was an error in **Table S1A concerning Rhizophagus irregularis *in planta* RNAseq libraries**.

The corrected **Table S1A** has been published as Supplementary material.

Accordingly, an addendum has been made to **Materials and Methods section, RNA Production and Sequencing, first paragraph:**

“Total RNA extraction and sequencing were performed according to Tisserant et al. (2013) for *R. irregularis* and Tang et al. (2016) for *G. rosea*. Apart from ERM of *G. rosea* where short paired- end sequencing reads were obtained from Illumina Miseq1000 protocols (2 × 151 bp), all libraries were obtained from Illumina Hiseq2000 protocols (2 × 101 bp). Library constructions and sequencing were performed on the GeT-PlaGe facility (Toulouse, France), according to standard Illumina protocols. Data are available at NCBI GEO portal (GSE67906 and GSE67911) for *G. rosea*, and at NCBI Sequence Read Archive (SRR1027885) and at NCBI GEO portal (GSE67926) (—see details on libraries in Table S1A) for *R. irregularis*. Number of reads per libraries, representativeness of fungal reads in symbiotic tissues and variability of data are presented in Tables S1A, S5A for *R. irregularis* and *G. rosea* respectively.”

The authors apologize for these errors and state that this does not change the scientific conclusions of the article in any way.

The original article has been updated.

## Conflict of interest statement

The authors declare that the research was conducted in the absence of any commercial or financial relationships that could be construed as a potential conflict of interest.

