# Supplementary figures and images for: The Comparison of Expressed Candidate Secreted Proteins from Two Arbuscular Mycorrhizal Fungi Unravels Common and Specific Molecular Tools to Invade Different Host Plants
Source: Front Plant Sci. 2017 Feb 7;8:124. doi: 10.3389/fpls.2017.00124 (PMC5293756; doi:10.3389/fpls.2017.00124)

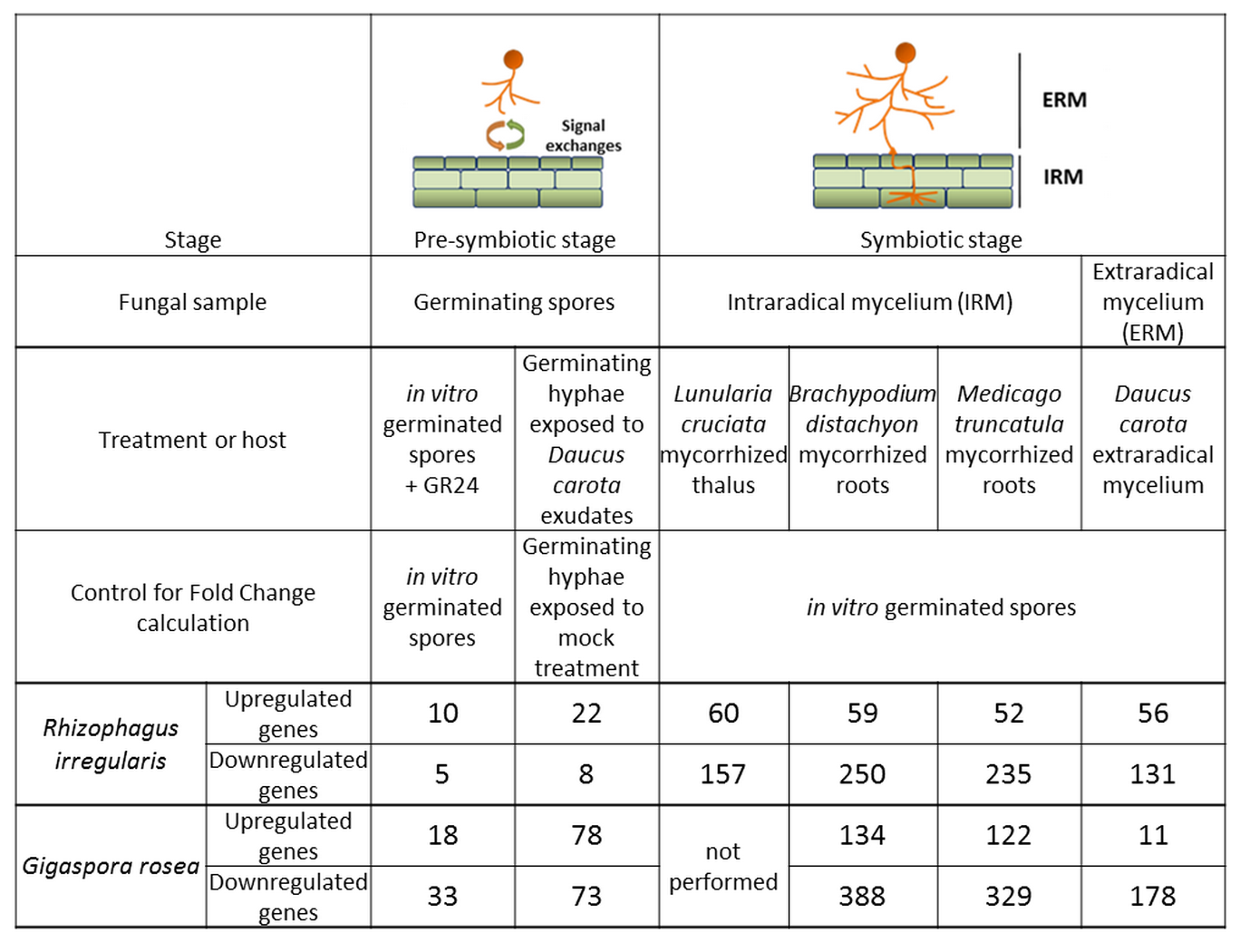

Supplement: Figure S1 — Overall presentation of the different biological conditions prepared for RNAseq comparisons. G. rosea and R. irregularis were both cultivated alone or in symbiosis with different hosts. Germinated spores in vitro (asymbiotic stage) were used as reference for all comparisons. See material and methods for details. FC, fold change. [file Image1.TIF]

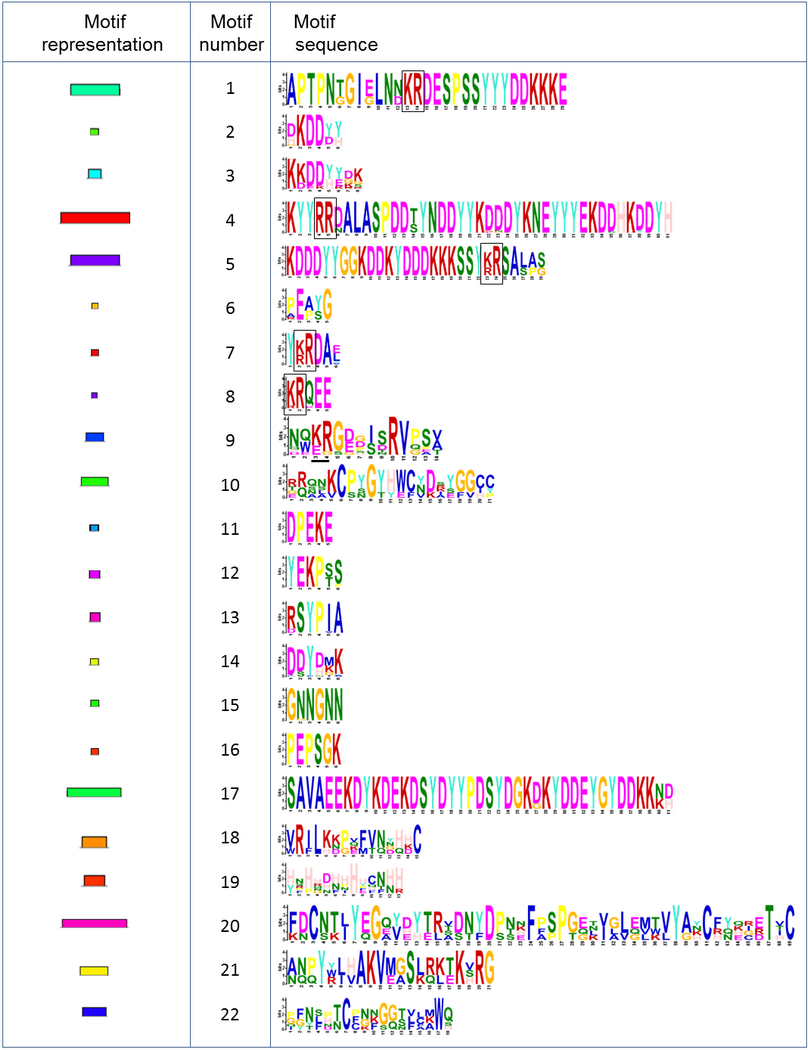

Supplement: Figure S2 — List of the motifs identified by the MEME discovery tool and presented in Figure 4. Boxes indicate putative KEX2 clivage sites ([KR]R). A non-canonical clivage site is underlined in Motif 9. [file Image2.TIF]

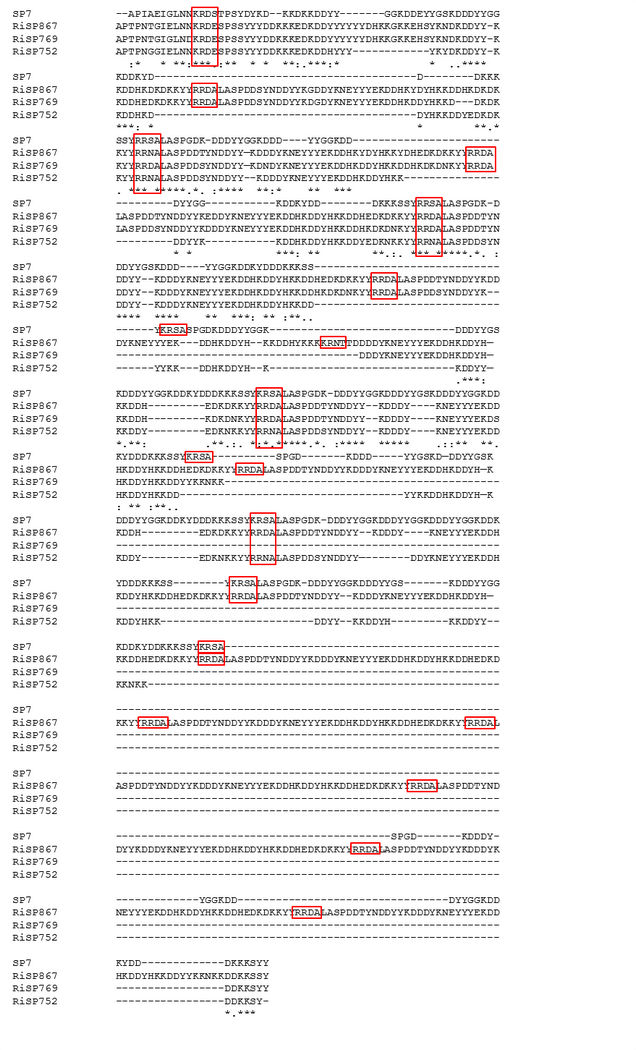

Supplement: Figure S3 — Sequence alignment of SP7 with three RiSPs displaying strong similarity. Alignment was made with MAFFT (v7.299b) and displayed with CLUSTAL format. Signal peptide was removed from the protein sequences. Boxes indicate putative clivage sites ([KR]R) followed by conserved amino acids. [file Image3.TIF]

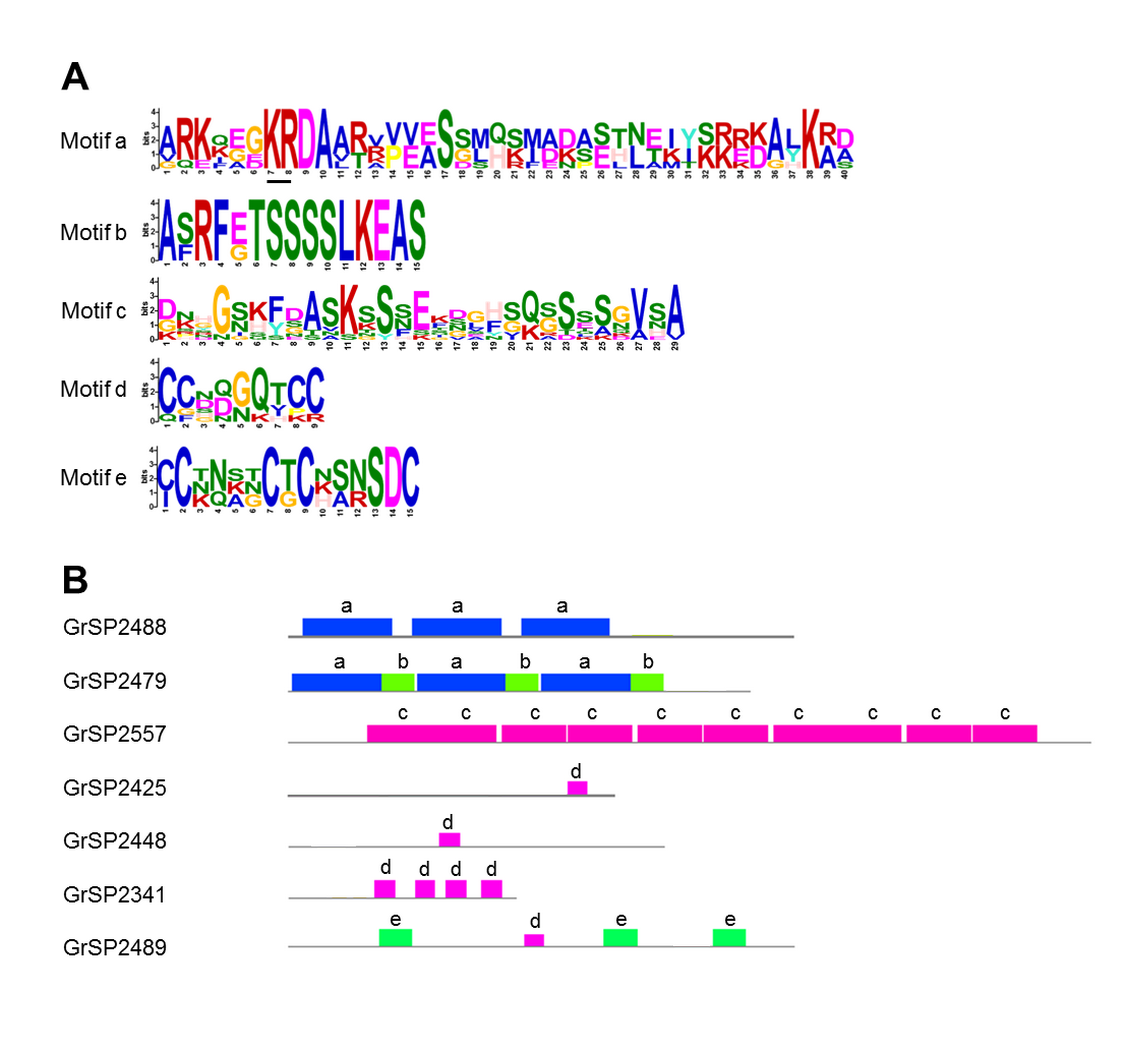

Supplement: Figure S4 — Protein structures (A) of GrSPs displaying motifs of unknown function (B). A putative KEX2 cleavage site is underlined in motif ≪ a ≫. [file Image4.TIF]

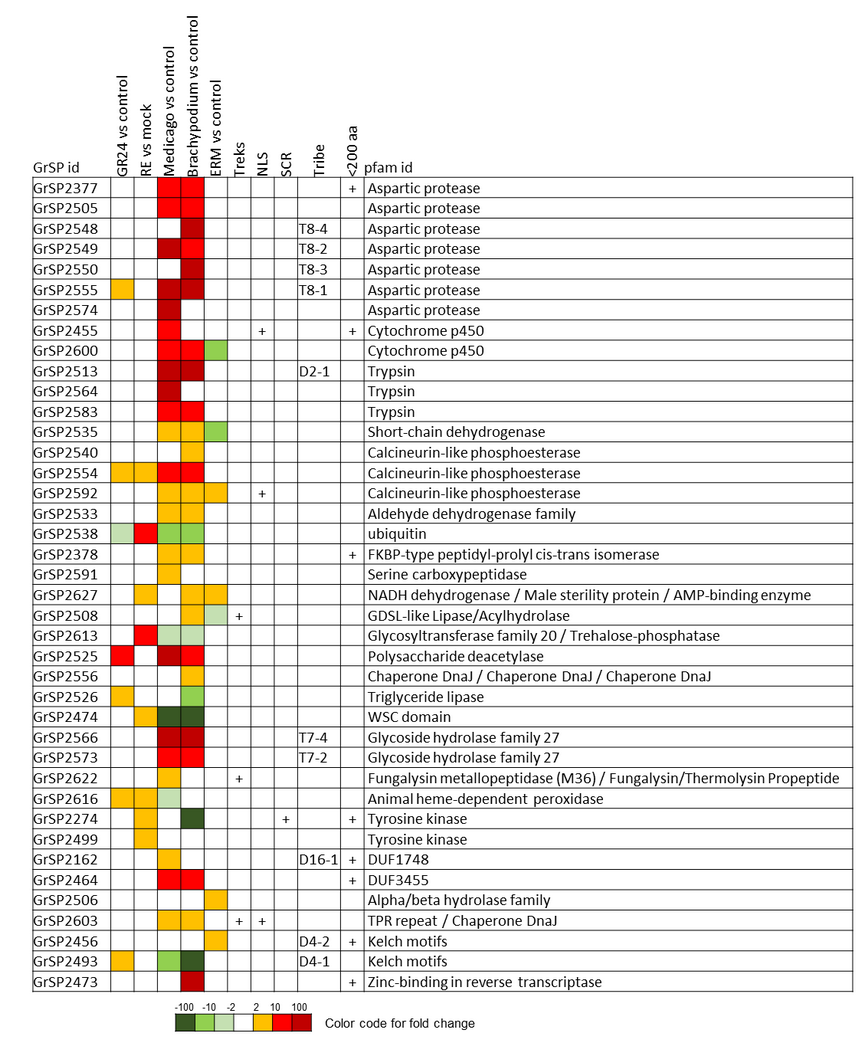

Supplement: Figure S5 — GrSPs with a PFAM domain up-regulated in at least one comparison. RE, root exudates; ERM, Extra-Radical Mycelium; NLS, Nuclear Localization Signal; SCR, Small Cysteine Rich. [file Image5.TIF]
